# The TrkB‐T1 receptor mediates BDNF‐induced migration of aged cardiac microvascular endothelial cells by recruiting Willin

**DOI:** 10.1111/acel.12881

**Published:** 2019-01-22

**Authors:** Zhefeng Wang, Yilin Chen, Xuwei Chen, Xin Zheng, Ganlin Xu, Ziqiang Yuan, Hui Zhao, Wensheng Chen, Lilin Li, Nianjue Zheng, Xiaotao Shen, Yanmei Li, Xufeng Qi, Dongqing Cai

**Affiliations:** ^1^ Key Laboratory of Regenerative Medicine, Ministry of Education Jinan University Guangzhou China; ^2^ Joint Laboratory for Regenerative Medicine Chinese University of Hong Kong‐Jinan University Guangzhou China; ^3^ International Base of Collaboration for Science and Technology (JNU), The Ministry of Science and Technology & Guangdong Province Guangzhou China; ^4^ Department of Developmental & Regenerative Biology Jinan University Guangzhou Guangzhou China; ^5^ Department of Medical Oncology Cancer Institute of New Jersey, Robert Wood Johnson of Medical School New Brunswick New Jersey; ^6^ Stem Cell and Regeneration TRP, School of Biomedical Sciences Chinese University of Hong Kong Hong Kong Hong Kong

**Keywords:** BDNF–TrkB, cardiac microvascular endothelial cell aging, Hippo pathway, migration, Willin/FRMD6

## Abstract

The mechanism of age‐related decline in the angiogenic potential of the myocardium is not yet fully understood. Our previous report revealed that the aging of cardiac microvascular endothelial cells (CMECs) led to changes in their expression of receptor Trk isoforms: among the three isoforms (TrkB‐FL, TrkB‐T1 and TrkB‐T2), only the truncated TrkB‐T1 isoform continued to be expressed in aged CMECs, which led to decreased migration of CMECs in aging hearts. Thus far, how BDNF induces signalling through the truncated TrkB‐T1 isoform in aged CMECs remains unclear. Here, we first demonstrated that aged CMECs utilize BDNF–TrkB‐T1 signalling to recruit Willin as a downstream effector to further activate the Hippo pathway, which then promotes migration. These findings suggest that the aging process shifts the phenotype of aged CMECs that express TrkB‐T1 receptors by transducing BDNF signals via the BDNF–TrkB‐T1–Willin–Hippo pathway and that this change might be an important mechanism and therapeutic target of the dysfunctional cardiac angiogenesis observed in aged hearts.

## 1 INTRODUCTION

Coronary artery disease‐induced myocardial infarction (MI) is one of the leading causes of morbidity and mortality among elderly individuals. Age‐related decline in the angiogenic potential of the myocardium is common among the elderly. Furthermore, the angiogenic factor‐mediated therapeutic effect, which is potent in young individuals, is less effective in older individuals. Thus, achieving functional regeneration following MI in the elderly remains a significant challenge (Ahluwalia, Jones, Szabo, & Tarnawski, 2014; Ahluwalia, Narula, Jones, Deng, & Tarnawski, 2010; Cai et al., 2003; Cao et al., 2012; Lähteenvuo & Rosenzweig, 2012). It is believed that endothelial cell aging shifts protective and angiogenic pathways, causing dysfunction in aged hearts, which may be an important aetiology of the age‐related decline in the regeneration of the myocardium and the poor cardiovascular prognoses observed in the elderly. However, the molecular mechanisms underlying these pathophysiologies remain unclear.

Cardiac microvascular endothelial cells (CMECs) play a central role in cardiac angiogenesis following MI due to their potent ability to initiate angiogenesis and to stimulate the secretion of multi‐angiogenic factors, such as vascular endothelial growth factor (VEGF) and platelet‐derived growth factor (PDGF), which act as autocrine and paracrine factors (Edelberg et al., 1998; Levéen et al., 1994). Brain‐derived neurotrophic factor (BDNF) and its receptor, TrkB, are expressed first during late gestation and then persistently at high levels into adulthood in the endothelial cells that line the arteries and capillaries of the heart. BDNF acts as an angiogenic factor, playing important roles in the survival of endothelial cells and angiogenesis (Donovan et al., 2000; Kermani & Hempstead, 2007). Compared with basic fibroblast growth factor (bFGF) alone, exogenous delivery of bFGF and BDNF to ischaemic myocardium improves the angiogenesis and left ventricular function of the ischaemic myocardium (Liu, Sun, Huan, Zhao, & Deng, 2006). BDNF is upregulated by neural signals in young hearts after MI and then protects the myocardium against ischaemic injury (Okada et al., 2012).

We previously reported that the number of truncated TrkB‐positive cells and the BDNF protein level are higher in the aged cardiac microvasculature. Furthermore, the BDNF level in aged ischaemic cardiac microvasculature is higher than the level in young nonischaemic, young ischaemic and old nonischaemic hearts. The age‐related increase in truncated TrkB in CMECs is apparently linked to an age‐associated increase in inflammatory responses and a significant increase in myocardial injury following MI (Cai et al., 2006). More recently, we found that BDNF can promote young CMECs to migrate via activation of the BDNF–TrkB‐FL–PI3K/Akt pathway, which may benefit angiogenesis after MI. However, the aging of CMECs leads to changes in the expression of isoforms of the receptor TrkB: Among the three isoforms (TrkB‐FL, TrkB‐T1 and TrkB‐T2), only the truncated TrkB‐T1 isoform continues to be expressed, which leads to dysfunction of its ligand, decreased CMEC migration and increased injury in aging hearts. This shift in receptor isoforms of the BDNF pathway in aged CMECs, together with changes in the aging microenvironment, might predispose aging hearts to decreased angiogenic potential and increased cardiac pathology. We also demonstrated that although the potency of promoting migration via the BDNF–TrkB‐T1 pathway in old CMECs was significantly decreased compared with young CMECs via the BDNF–TrkB‐FL pathway, the BDNF–TrkB‐T1 pathway in old CMECs can still promote the migration of aged CMECs (Cao et al., 2012). The full‐length TrkB receptor (TrkB‐FL) undergoes autophosphorylation to activate intracellular signalling pathways (Gupta, You, Gupta, Klistorner, & Graham, 2013). Furthermore, so far knowledge suggested that TrkB‐T1 is recognized as a dominant negative receptor that inhibits TrkB‐FL signalling, as it lacks the catalytic tyrosine kinase domain (Fenner, 2012). However, it remains to be determined how aged CMECs transduce BDNF signalling to regulate effects via the BDNF–TrkB‐T1 pathway under physiopathological situations. Our previous findings led us to hypothesize that aging causes a shift in the phenotype of BDNF receptors in old CMECs, which only express TrkB‐T1 and not TrkB‐FL, to transduce BDNF signals via BDNF–TrkB‐T1 activity to mitigate physiopathological homogenesis and compensate for the declining cardiovascular system. Aged CMECs might play a role in an unknown mechanism to transduce BDNF signals via the BDNF–TrkB‐T1 pathway to recruit unknown downstream effectors to maintain the angiogenic potential and capacity of the heart. In this study, we first report a novel mechanism by which aged CMECs expressing the TrkB‐T1 receptor isoform recruit Willin/FRMD6 as a downstream effector to activate the Hippo‐Yap pathway, thus transducing the BDNF signal to promote their migration.

## 2 RESULTS

### 2.1 Identification of Willin/FRMD6 as a novel TrkB‐T1 interaction partner in old CMECs

To screen for downstream effectors of TrkB‐T1 in old CMECs, a library of old rat CMEC cDNA was constructed for yeast two‐hybrid screening, as TrkB‐T1 is only expressed in old CMECs. The pGBKT7‐T1‐ICD bait vector including the full intracellular TrkB‐T1 domain was constructed and applied as bait to screen the established old cDNA library of CMECs. We first tested the autoactivation activity of the bait proteins in yeast cells. The pGBKT7‐T1‐ICD and pGBKT7‐T1‐C11 bait plasmids were transformed into Y2HGold cells, and the transformants were grown on SD/‐Trp, SD/‐Trp/X‐α‐Gal and SD/‐Trp/X‐α‐Gal/AbA (Supporting Information Figure S1I). The results showed that no autoactivation activity was detected from pGBKT7‐T1‐ICD or pGBKT7‐T1‐C11, as shown in Supporting Information Figure S1I.

To screen host proteins that interacted with the pGBKT7‐T1‐ICD bait by the Y2H system, Y2HGold cells containing the pGBKT7‐T1‐ICD plasmid were used to mate with Y187 cells harbouring the established old cDNA library of CMECs. The fifteen blue colonies were grown on SD/‐Leu/‐Trp/‐His/‐Ade/+X‐α‐Gal/AbA agar plates (Supporting Information Figure S1II). The specific insert on each blue prey colony was sequenced. Three validated sequences named Prey A, Prey B and Prey C, were identified (Supporting Information Figure S1III). To exclude the autoactivation activity of Prey A, Prey B and Prey C, the Y187 strain transformed separately with Prey A, Prey B and Prey C plasmid was used to mate with Y2HGold containing pGBKT7‐T‐BD plasmid (empty pGBKT7 plasmid). No blue colony grew on SD/‐Leu/‐Trp/‐His/‐Ade/+X‐α‐Gal/AbA agar plates (Supporting Information Figure S1IV). To investigate whether the interaction site of TrkB‐T1 with prey was located in the C‐terminus, strain Y187 transformed separately with Prey A, Prey B and Prey C plasmid was used to mate with Y2HGold containing pGBKT7‐T1‐C11 bait plasmid (including 11 C‐terminal amino acids of the TrkB‐T1 intracellular domain) and pGBKT7‐T1‐ICD bait plasmid. The results with pGBKT7‐T1‐ICD showed that all of the zygotes resulting from mating turned blue on SD/‐Leu/‐Trp/‐His/‐Ade/+X‐α‐Gal/AbA agar plates (Supporting Information Figure S1V), while the results with pGBKT7‐T1‐C11 showed that the zygotes resulting from mating with Prey A and Prey C turned blue on SD/‐Leu/‐Trp/‐His/‐Ade/+X‐α‐Gal/AbA agar plates (Supporting Information Figure S1V). These data suggest that the interaction site between TrkB‐T1 and Prey A and Prey C was located within those 11 amino acids of the C‐terminus of TrkB‐T1.

Three candidate proteins that potentially interacted with TrkB‐T1 were revealed via a blast homology analysis of the screened DNA sequence (Supporting Information Figure S1III). Prey A, which was homologous with Willin, also named FRMD6, was selected for further study, as it was the only cytosolic protein among the three screened candidates (Prey B was a membrane protein and Prey C was a nuclear protein) (Supporting Information Figure S1VI).

**Figure 1:**
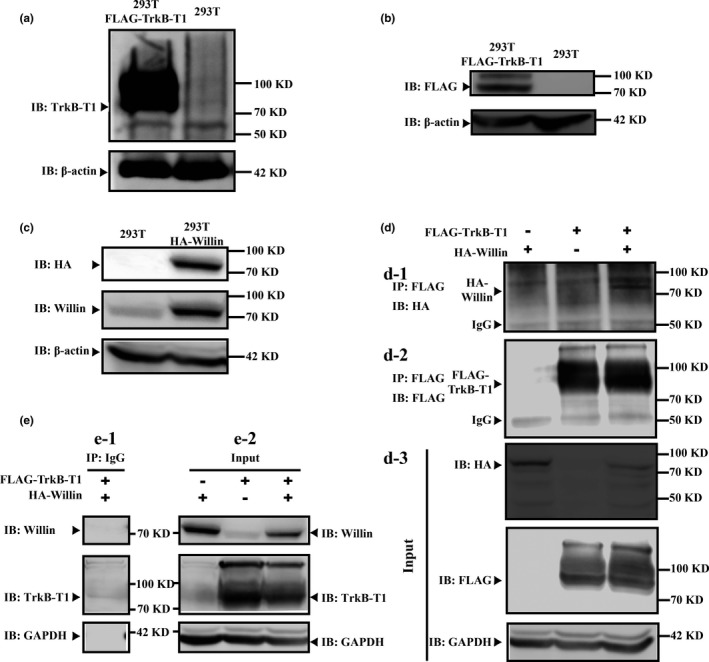
Co‐IP confirms the interaction between TrkB‐T1 and Willin. (a) Western blot (WB) revealed that wild‐type 293T cells did not express TrkB‐T1, while TrkB‐T1‐FLAG‐tag‐transfected 293T cells highly expressed TrkB‐T1‐FLAG‐tag. (b) WB confirmed that TrkB‐T1 was expressed ectopically in TrkB‐T1‐FLAG‐tag 293T cells. (c) WB confirmed that Willin was expressed ectopically in Willin‐HA‐tag 293T cells and wild‐type 293T cells expressed Willin. (d) The TrkB‐T1–Willin interaction was only detectable when Willin‐HA‐tag and TrkB‐T1‐FLAG‐tag plasmids were cotransfected in the 293T cells (d‐1; arrow). TrkB‐T1‐FLAG is precipitated specifically (d‐2: arrow). Input lysis WB demonstrated that TrkB‐T1‐FLAG‐tag and Willin‐HA‐tag were included alone or together in transfected lysis (d‐d3; e‐e2). (e) The used FLAG‐Co‐IP system for TrkB‐T1 precipitated the FLAG‐TrkB‐T1 specifically

Direct interaction between TrkB‐T1 and Willin was validated by co‐immunoprecipitation (Co‐IP) and western blot. As 293 T cells did not express TrkB‐T1 receptor (Figure 1a), they represented a suitable model for investigating the interaction between TrkB‐T1 and Willin using an ectopic expression strategy, which would exclude possible cross‐effects from endogenous TrkB‐T1 receptor. We first confirmed that the TrkB‐T1‐FLAG‐tag and Willin‐HA‐tag were expressed ectopically in 293 T cells after transfection (Figure 1b and c). In addition, 293 T cells expressed Willin, and the Willin in 293 T cells transfected with Willin‐HA‐tag was upregulated significantly (Figure 1c). Then, we further found that the TrkB‐T1–Willin interaction was only detectable when plasmids encoding Willin‐HA‐tag and plasmids encoding TrkB‐T1‐FLAG‐tag were cotransfected in the 293 T cells and not in cells transfected with plasmids encoding the FLAG‐TrkB‐T1 control or plasmids encoding the Willin‐HA‐tag control, as detected by an anti‐HA Co‐IP elution western blot (Figure 1d). Input lysis western blot (anti‐HA, anti‐FLAG, anti‐Willin and anti‐TrkB‐T1) demonstrated that TrkB‐T1‐FLAG‐tag and Willin‐HA‐tag were included alone or together in transfected lysis (Figure 1d‐d3 and e‐e2). Co‐IP control assay for anti‐IgG confirmed that the used FLAG‐Co‐IP system for TrkB‐T1 was able to precipitate the FLAG‐TrkB‐T1 specifically (Figure 1e‐e1). All these suggest that TrkB‐T1 is able to interact with Willin directly.

**Figure 2:**
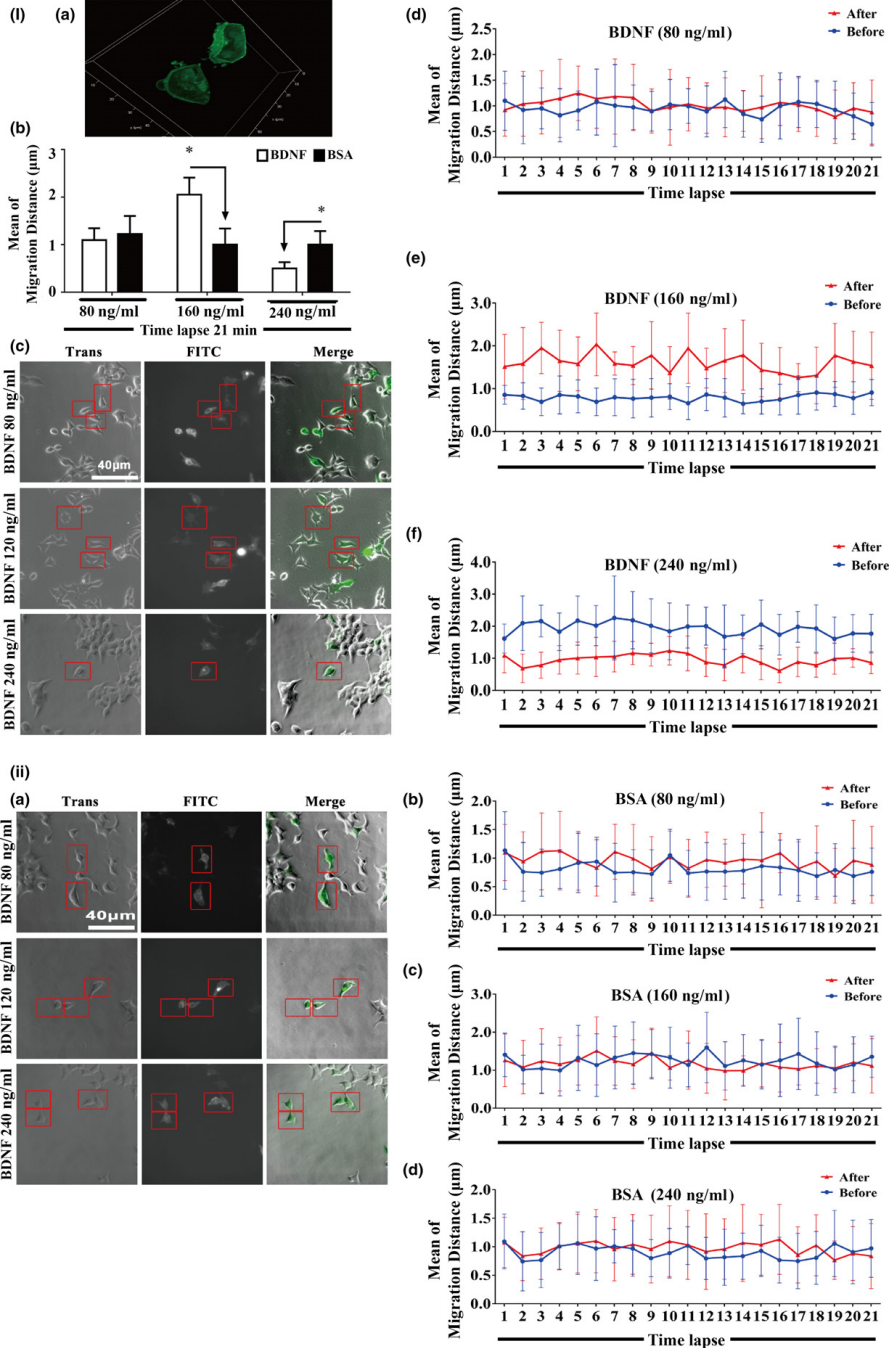
BDNF–TrkB‐T1–Willin pathway promotes the activity of pseudopods in TrkB‐T1‐transfected 293T cells. I: (a) Ectopically expressed TrkB‐T1‐EGFP in the 293T cells was localized in the cell membrane. (b) Semiquantitative analysis of migration distance of pseudopods of TrkB‐T1‐EGFP‐transfected positive 293T cells which were treated with BDNF and BSA (80, 160 and 240 ng/ml). (*p < 0.05 vs. BSA). (c) Representative images of TrkB‐T1‐EGFP‐transfected 293T cells treated with different doses of BDNF (80, 160 and 240 ng/ml). (d‐f) A representative measurement of pseudopod migration in cells of (c). II: (a) Representative images of TrkB‐T1‐EGFP‐transfected 293T cells treated with different doses of BSA (80, 160 and 240 ng/ml). (b‐d) A representative measurement of pseudopod migration in cells of (a). The time‐lapse analysis demonstrated that BDNF (160 ng/ml, but not 80 ng/ml or 240 ng/ml) significantly increased the migration distance of pseudopods of the TrkB‐T1‐EGFP‐positive cells compared with BSA control. Red rectangle indicates the measured TrkB‐T1‐transfected positive cells. Bar = 40 μm

To observe the TrkB‐T1 subcellular localization and interaction with Willin in living cells, we employed bimolecular fluorescence complementation (BiFC). We engineered TrkB‐T1 and Willin fusions to VC155 and VN173 of EGFP, which are derivatives of green fluorescent protein produced when VC155‐TrkB‐T1 and VN173‐Willin interact with each other (Supporting Information Figure S2I). A significantly higher fluorescence density was found in the TrkB‐T1‐VC155‐ and Willin‐VN173‐cotransfected 293 T cell group but not in the TrkB‐T1‐VC155‐, Willin‐VN173‐, VC‐155‐, or VN173‐transfected 293 T cell group. The fluorescence density of the TrkB‐FL‐VC155‐ and Willin‐VN173‐cotransfected 293 T cell group was significantly lower than the TrkB‐T1‐VC155‐ and Willin‐VN173‐cotransfected 293 T cell group (Supporting Information Figure S2II; *p* < 0.01). In addition, most of the green fluorescence was located in the cell membranes in the TrkB‐T1‐VC155‐ and Willin‐VN173‐cotransfected group (Figure 2Ia). However, the differences in the green fluorescence density between the TrkB‐T1‐VC155‐, Willin‐VN173‐, VC‐155‐, and VN173‐transfected 293 T cell groups were not statistically significant (Supporting Information Figure S2II; *p* > 0.05). Furthermore, a competitive BiFC strategy using TrkB‐T1‐FLAG‐tag and Willin‐HA‐tag as competitors for TrkB‐T1‐VC155 and Willin‐VN173, respectively, also confirmed that the fluorescence density decreased significantly when the TrkB‐T1‐FLAG‐tag expression vector or the Willin‐HA‐tag expression vector was transfected in the TrkB‐T1‐VC155‐ or Willin‐VN173‐transfected group compared with the TrkB‐T1‐VC155‐ and Willin‐VN173‐cotransfected group (Supporting Information Figure S2III; *p* < 0.01). This indicated that the intracellular domain of TrkB‐T1 interacts with Willin much more strongly than TrkB‐FL dose. The critical amino acids for the interaction between the intracellular domain of TrkB‐T1 and Willin were further screened by homology modelling combined with bioinformatics analysis. The homology modelling analysis predicted that the intracellular domain loop of TrkB‐T1 was included in structural pocket of the FERM domain of Willin (Supporting Information Figure S3). The predicted interacting amino acids between the TrkB‐T1 intracellular domain and the FERM domain of Willin were GLY23‐ARG227 (TrkB‐T1 residues–Willin residues), ‐ARG301, ASP22‐TYR69, and others via interface nonbonding interactions (Table S1), while ASP22‐TYR69, ‐CYS298 and others interacted via hydrogen bonds (Table S2). The above results combined with the result of Supporting Information Figure S1V suggest that the 11 C‐terminal amino acids of the TrkB‐T1 intracellular domain are the binding domain between TrkB‐T1 and the FERM domain of Willin.

### 2.2 BDNF–TrkB‐T1–Willin pathway promotes the activity of pseudopods

**Figure 3:**
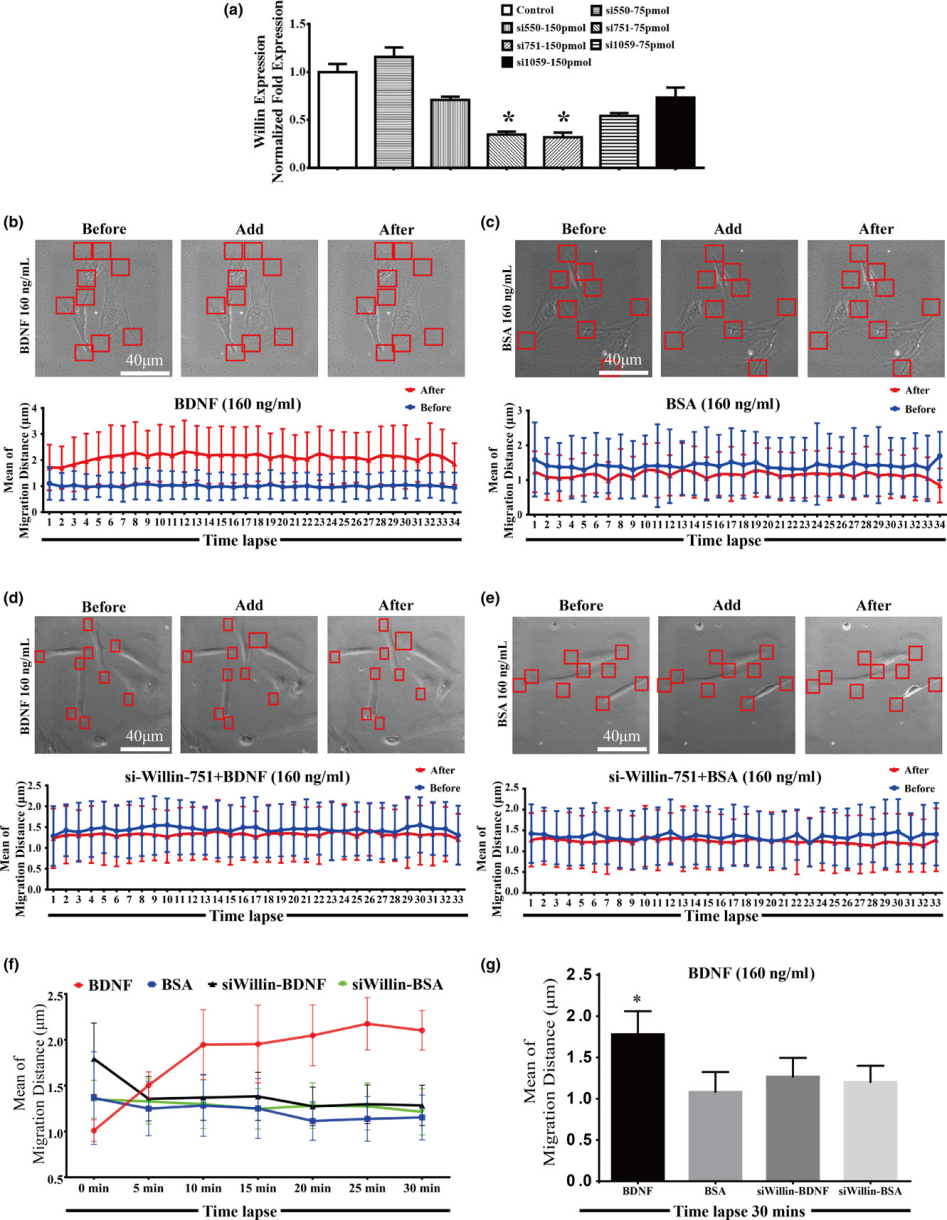
BDNF–TrkB‐T1–Willin pathway promotes the activity of pseudopods in old CMECs. (a) si‐Willin‐751 was applied, as it induced the strongest knockdown effect of Willin (*p < 0.05 for si751–75 pmoL and si751–150 pmoL vs. other groups respectively). (b–c) Representative images of BDNF (160 ng/ml)‐ and BSA (160 ng/ml)‐treated old CMECs and a representative measurement of pseudopod migration. (d–e) Representative images of BDNF (160 ng/ml)‐treated old CMECs under si‐Willin‐751 transfection and BSA (160 ng/ml)‐treated old CMECs under si‐Willin‐751 transfection, and a representative measurement of pseudopod migration. (f and g) Semiquantitative analysis of b, c, d and e. The time‐lapse analysis demonstrated that BDNF (160 ng/ml) increased the migration distance of pseudopod of CMECs compared with BSA control (*p* < 0.05 vs. 5‐, 10‐, 15‐, 20‐, 25‐, 30‐min). Furthermore, knockdown of Willin using si‐Willin‐751 in old CMECs abrogated the effect of BDNF of increasing the migration distance (*p* > 0.05 for si‐Willin‐751‐BDNF vs. BSA, si‐Willin‐751‐BSA). (f) Analysis shown as time‐lapse pattern. (g) Analysis of mean migration distance for 30‐min time lapse (*p < 0.05 vs. other groups). Bar = 40 μm. Red rectangles in b, c, d and e indicate the locations of measured cells

Our previous findings documented that BDNF is able to promote the migration of old CMECs which only express TrkB‐T1 receptor and not TrkB‐FL (Cao et al., 2012). Based on the TrkB‐T1 yeast two‐hybrid screen with old rat CMEC cDNA and the observation that TrkB‐T1 interacted with Willin, we further proposed that the BDNF–TrkB‐T1 pathway might recruit Willin as a downstream effector to transduce BDNF signalling in old CMECs. As promoting the activity of pseudopods is one of the initiating steps for promoting the migration of cells, we applied a time‐lapse technique to test whether the BDNF–TrkB‐T1–Willin pathway promoted the activity of pseudopods. We again applied 293 T cells, which did not express TrkB‐T1 but did express Willin (Figure 1a and c), to induce the BDNF–TrkB‐T1–Willin pathway via ectopic expression of TrkB‐T1‐EGFP. We first confirmed that the ectopically expressed TrkB‐T1 in these cells was located in the cell membrane (Figure 2Ia). Our time‐lapse study demonstrated that BDNF (160 ng/ml, but not 80 ng/ml or 240 ng/ml) significantly promoted the migration distance of the pseudopod of the TrkB‐T1‐EGFP‐positive cells compared with the BSA control. Since the effect of BDNF was dose‐dependent, this dose of BDNF (160 ng/ml) was applied in the following experiments (Figure 2I and II; *p* < 0.05). In addition, knockdown of Willin using si‐Willin‐2095 siRNA, which was confirmed to effectively downregulate Willin (Supporting Information Figure S4Ia; *p* < 0.05), abrogated the lengthening of the migrating distance of BDNF in TrkB‐T1‐EGFP‐positive cells (Supporting Information Figure S4I and II; *p* > 0.05). This suggested that BDNF–TrkB‐T1 recruited Willin to facilitate the activity of the pseudopod. Next, we further confirmed this effect in old CMECs. We first confirmed that si‐Willin‐751 best knocked down Willin expression in old CMECs (Figure 3a; *p* < 0.05). We also demonstrated that BDNF (160 ng/ml) lengthened the migration distance of the pseudopod of CMECs compared with the BSA control (Figure 3b,c,f and g; *p* < 0.05). In addition, knockdown of Willin using si‐Willin‐751 in old CMECs abrogated the effect of BDNF of increasing the migrating distance (Figure 3d–f and g; *p* > 0.05). Furthermore, under the same dose of BDNF treatment (160 ng/ml), the mean migration distance of old CMECs was similar to that of TrkB‐T1‐transfected 293 T cells (Figures 2Ib and 3g). These results suggest that the BDNF–TrkB‐T1–Willin pathway in old CMECs facilitated the migration of pseudopods.

### 2.3 BDNF–TrkB‐T1–Willin pathway increases the polarity of actin and polymerization of stress fibres

The polarity of actin and the polymerization of stress fibres are crucial for initiating mobility potential. Therefore, we further tested whether the BDNF–TrkB‐T1–Willin pathway is able to increase the polarity of actin and the polymerization of stress fibres. We performed a time‐lapse experiment combined with *F*‐actin immunofluorescence to analyse these parameters in old CMECs. The number of polarized cells was progressively increased when observed at 5 min, 15 min and 30 min after BDNF treatment (160 ng/ml). The percentage of polarized cells was 81.03% ± 0.03% at 5 min and reached 90.08 ± 0.03% at 30 min after BDNF treatment. However, the percentages of polarized cells in the BSA‐treated groups at parallel time points were similar to each other (Supporting Information Figure S5I; *p* > 0.05). In addition, the mean diameter of stress fibres in the BDNF‐treated group was significantly larger than that of the BSA‐treated group 30 min after BDNF treatment (Supporting Information Figure S5II; *p* < 0.05). Together, these results suggest that the BDNF–TrkB‐T1–Willin pathway increased the polarity of actin and the polymerization of stress fibres in BDNF‐treated old CMECs.

### 2.4 BDNF–TrkB‐T1–Willin pathway promotes the migration of old CMECs

**Figure 4:**
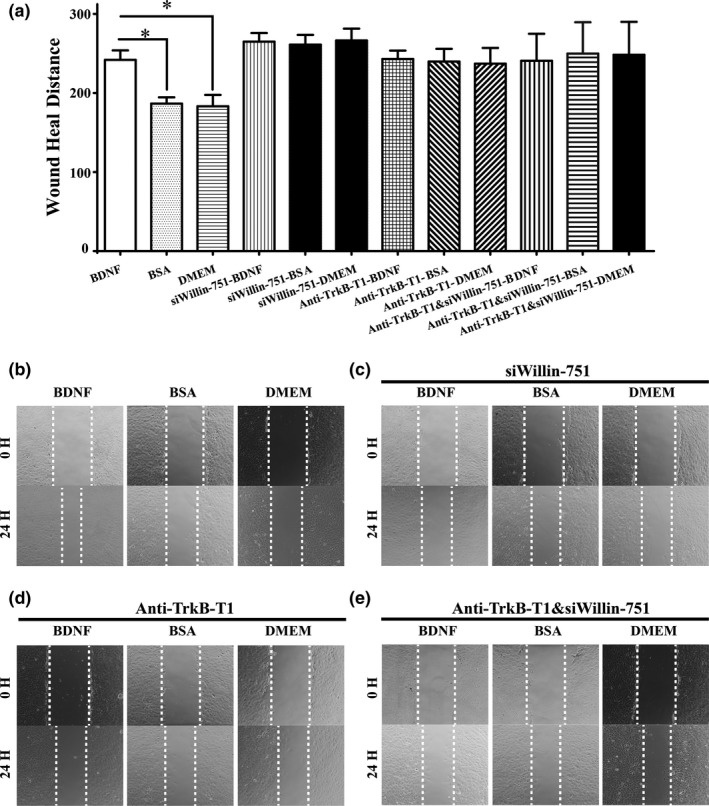
The BDNF–TrkB‐T1–Willin pathway promotes the migration of old CMECs. (a) Semiquantitative analysis of the migration in different groups based on the scratch assay. (b) Representative images of BDNF‐, BSA‐ and DMEM‐treated old CMECs. (c) Representative images of BDNF‐, BSA‐ and DMEM‐treated old CMECs that were pretransfected with si‐Willin‐751. (d) Representative images of BDNF‐, BSA‐ and DMEM‐treated old CMECs that were pretreated with an anti‐TrkB‐T1 antibody. (e) Representative images of BDNF‐, BSA‐ and DMEM‐treated old CMECs that were pretransfected with si‐Willin‐751 and pretreated with an anti‐TrkB‐T1 antibody. The migration distance of old CMECs in the BDNF‐treated group was significantly longer than those of the BSA‐ and DMEM‐treated controls (*p < 0.05 vs. BSA and DMEM). The differences in the migration distances after treatment with BDNF in the anti‐TrkB‐T1‐pretreated group, si‐Willin‐751‐transfected group and anti‐TrkB‐T1+si‐Willin‐751‐transfected group were not statistically significant compared with the respective BSA and DMEM control groups (*p* > 0.05)

The above evidence clearly demonstrated that the BDNF–TrkB‐T1–Willin pathway promoted the migration potential of old CMECs. We therefore applied an in vitro scratch wound healing assay to investigate whether the BDNF–TrkB‐T1–Willin pathway is able to promote the migration of old CMECs. We confirmed that the migration distance of old CMECs in the BDNF‐treated group was significantly longer than that of the controls (Figure 4a and b; *p* < 0.05), while the differences in the migration distances after treatment with BDNF in the anti‐TrkB‐T1‐pretreated group, the si‐Willin‐751‐transfected group and the anti‐TrkB‐T1+si‐Willin‐751‐transfected group were not statistically significant compared with the respective BSA and DMEM control groups (Figure 4a,c,d and e; *p* > 0.05). The results revealed that the BDNF–TrkB‐T1–Willin pathway promoted the migration of old CMECs, and the increased migration effect in old CMECs after BDNF treatment was attributed to binding with TrkB‐T1 receptors, which led to the recruitment of the downstream effector, Willin.

### 2.5 The BDNF–TrkB‐T1–Willin pathway promotes the migration of old CMECs via Downstream activation of hippo signalling

**Figure 5:**
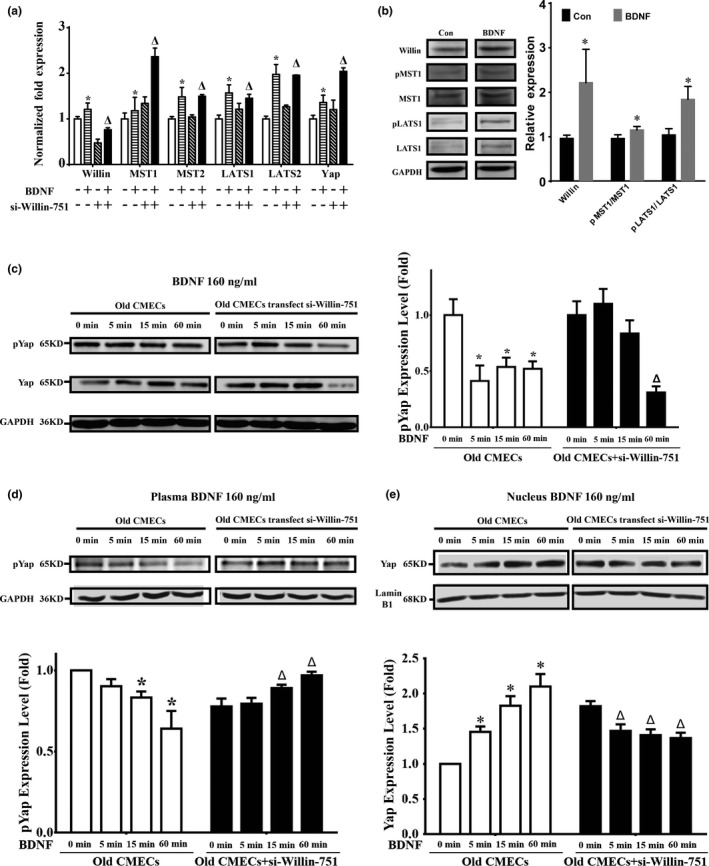
BDNF–TrkB‐T1–Willin pathway promotes the migration activity of old CMECs via downstream activation of Hippo signalling. (a) qPCR showed that BDNF upregulated *Willin*,* MST1*,* MST2*,* LATS1, LATS2* and *Yap* in old CMECs (*p < 0.05 vs. non‐BDNF+non‐si‐Willin‐751‐control). In addition, upregulated expression patterns of *MST1*,* LATS1*,* LATS2* and *Yap* in the si‐Willin‐751‐treated and si‐Willin‐751+BDNF‐treated old CMECs compared with the old CMEC group were found (△: *p* < 0.05 vs. si‐Willin‐751). (b) BDNF treatment increased Willin expression and promoted the phosphorylation of MST1/2 and LATS1/2 in old CMECs. (c) BDNF treatment decreased the phosphorylation level of Yap in whole‐cell lysates of old CMECs from 5 to 60 min after BDNF treatment. However, knockdown of Willin by si‐Willin‐751 delayed the decrease in Yap phosphorylation (*p < 0.05 vs. 0 min). (d) Compared with non‐BDNF‐treated cells, the level of phosphorylated Yap in the cytoplasm was decreased from 5 to 60 min after BDNF treatment. However, when Willin was knocked down by si‐Willin‐751 in old CMECs, BDNF treatment induced a progressive increase in phosphorylated Yap in the cytoplasm (*p < 0.05 vs. other groups; △*p* < 0.05 vs. other groups.). (e) The level of dephosphorylated Yap in the nucleus of old CMECs was increased from 5 to 60 min after BDNF treatment (*p < 0.05 vs. other groups). However, when Willin expression was knocked down by si‐Willin‐751, BDNF treatment for 60 min caused a decrease in dephosphorylated Yap in the nuclei (△*p* < 0.05 vs. 0 min). (a–e) Results suggest that in old CMECs, BDNF–TrkB‐T1–Willin signalling activated the Hippo pathway

Willin is an upstream component of the Hippo signal pathway; therefore, Hippo signal molecules (MST1/2, LAST1/2 and Yap) (Angus et al., 2012; Moleirinho et al., 2013) were further investigated in old CMECs. Our qPCR results showed that BDNF significantly upregulated the mRNAs of *Willin*,* MST1*,* MST2, LATS1*,* LATS2* and *Yap* genes in old CMECs (Figure 5a; *p* < 0.05). si‐Willin‐751 transfection in old CMECs knocked down *Willin*, and under si‐Willin‐751 knockdown and BDNF treatment, *MST1*,* MST2, LATS1*,* LATS2* and *Yap* were upregulated compared with the non‐Willin‐751 + non‐BDNF‐treated group (Figure 5a; *p* < 0.05). Importantly, BDNF promoted the phosphorylation of MST1/2 and LATS1/2 and the expression of Willin (Figure 5b; *p* < 0.05). These results suggest that in old CMECs, BDNF treatment upregulated and activated key effectors of the Hippo pathway via the BDNF–TrkB‐T1–Willin pathway. Furthermore, the results also suggest that as the downstream effectors of *Willin*, the expression levels of *MST1*,* MST2*,* LATS1*,* LATS2* and *Yap* sensed the downregulation of *Willin* after si‐Willin‐751 treatment and subsequently upregulated themselves as a feedback response to maintain the pathway activity in vivo. In addition, Willin, MST1, MST2, LATS1, LATS2 and Yap were expressed in both young and old CMECs. At the mRNA level, the differences in the expression levels of *Willin, MST1, MST2, LATS2 and Yap* between young CMECs and old CMECs were not statistically significant (*p* > 0.05), while the expression of *LATS1* in young CMECs was significantly higher than that in old CMECs (Supporting Information Figure S6a; *p* < 0.05). However, the western blot results confirmed that the differences in expression of Willin, MST1, LATS1 and Yap between young and Old CMECs were not statistically significant at the protein level (Supporting Information Figure S6b; *p* > 0.05). This suggests that old CMECs only expressing TrkB‐T1 might be a key reason why old CMECs recruit Willin to activate the Hippo pathway to transduce the BDNF signal to promote their migration.

As the dephosphorylation of Yap and its translocation into nuclei are a hallmark of activation of the Hippo pathway (Zeng & Hong, 2008), we observed the dynamic changes in phosphorylated Yap in the cytoplasm and nuclei by performing western blots for proteins from the nuclear and cytoplasmic fractions of BDNF‐treated old CMECs. We first found that BDNF treatment decreased the phosphorylation level of Yap in whole‐cell lysates at 5 min to 60 min after BDNF treatment (Figure 5c; *p* < 0.05). However, knockdown of Willin expression by si‐Willin‐751 delayed the dephosphorylation of Yap. The phosphorylation level of Yap in the si‐Willin‐751‐treated old CMECs was still higher at 15 min after BDNF treatment compared with the same time point of BDNF‐treated old CMECs (Figure 5c; *p* > 0.05), which suggested that the BDNF–TrkB‐T1–Willin pathway promoted the dephosphorylation of Yap in old CMECs. The Yap localization between the cytoplasm and nucleus under BDNF‐ and non‐BDNF‐treated conditions was further investigated in old CMECs. Compared with the non‐BDNF treatment condition, the level of phosphorylated Yap in the cytoplasm was decreased from 5 min to 60 min after BDNF treatment (Figure 5d; *p* < 0.05). However, when Willin was knocked down by si‐Willin‐751 in old CMECs, BDNF treatment induced a progressive increase in the level of phosphorylated Yap in the cytoplasm (Figure 5d; *p* < 0.05). In parallel, the level of dephosphorylated Yap in the nucleus of old CMECs was increased from 5 min to 60 min after BDNF treatment (Figure 5e; *p* < 0.05). Furthermore, when Willin was knocked down by si‐Willin‐751 in old CMECs, BDNF treatment for 60 min caused a decrease in the level of dephosphorylated Yap in the nucleus (Figure 5e; *p* < 0.05). These results reveal that in old CMECs, BDNF treatment for approximately 60 min induced a decrease in the level of Yap phosphorylation in the cytoplasm and an increase in the level of Yap dephosphorylation in the nucleus, while knocking down Willin expression in old CMECs abrogated the BDNF‐mediated decrease in Yap phosphorylation in the cytoplasm and increased Yap dephosphorylation in the nucleus.

In addition, immunofluorescence staining for Yap was applied to observe the dynamic change in Yap localization in old CMECs in situ after BDNF treatment. It was revealed that BDNF treatment for 30 min induced an increase in the percentage of equal‐phosphorylation‐density cells (meaning the fluorescence intensity of the cytoplasm equalled that of the nucleus, as shown in Supporting Information Figure S7Ic) to approximately 83.65%, whereas the percentage was approximately 44.32% in the non‐BDNF‐treated cells (Supporting Information Figure S7II). In the BSA control group treated for 30 min, the percentage of equal‐phosphorylation‐density cells was approximately 37.18%, which was similar to the non‐BDNF‐treated case (approximately 30.59%) (Supporting Information Figure S7III). In addition, in the si‐Willin‐751‐treated group, the percentage of equal‐phosphorylation‐density cells after BDNF treatment for 30 min (approximately 27.05%) was lower than that of the non‐BDNF‐treated group (approximately 45.61%) (Supporting Information Figure S7IV), while the percentage of equal‐phosphorylation‐density cells after BSA treatment for 30 min (approximately 43.14%) was similar to the non‐BSA‐treated group (approximate 37.50%) (Supporting Information Figure S7V). This result further confirmed that BDNF treatment induced the translocation of dephosphorylated Yap into the nucleus via the BDNF–TrkB‐T1–Willin pathway. The above findings clearly demonstrate that BDNF treatment of aged CMECs induces dephosphorylation of Yap in the cytoplasm and induces the dephosphorylated Yap to migrate into the nuclei.

## 3 DISCUSSION

In this study, using the yeast two‐hybrid system, we screened Willin/FRMD6 as a candidate downstream recruited effector of TrkB‐T1. The results of the Co‐IP assay confirmed that Willin is able to bind with the intracellular domain of TrkB‐T1, and the results of the BiFC and competing BiFC assays also verified that the intracellular domain of TrkB‐T1 interacts with Willin with a high affinity and not randomly. All these results reveal that Willin interacts with the intracellular domain of TrkB‐T1 and may act as a downstream effector that is recruited by TrkB‐T1. Furthermore, the BiFC and competing BiFC assays showed that Willin is able to interact strongly with TrkB‐T1 but not TrkB‐FL in vivo. This indicates that recruitment of Willin as a downstream molecule is unique to TrkB‐T1 and not TrkB‐FL. In addition, the yeast mating assay between the 11 C‐terminal amino acids of TrkB‐T1 bait (Y2HGold containing pGBKT7‐T1‐C11 bait plasmid) and Willin (Prey A) revealed that TrkB‐T1 recruits Willin as a downstream effector via its intracellular domain of 11 amino acids of the C‐terminus. Furthermore, the results of homology modelling analysis further revealed that the 11 C‐terminal amino acids of TrkB‐T1 (intracellular domain) are the binding domain between TrkB‐T1 and the FERM domain of Willin. All these data clearly show that TrkB‐T1 receptor is able to recruit Willin via its specific 11 C‐terminal amino acids to interact with the FERM domain of Willin, but this does not happen for TrkB‐FL, which does not have the relevant domain. In support of this finding, the FERM domain of Willin is sufficient to activate the Hippo pathway via MST1/2 and to antagonize Yap‐induced phenotypes in mammalian cells (Angus et al., 2012).

To address whether Willin is involved in BDNF–TrkB‐T1‐mediated migration of aged CMECs, we applied 293 T cells, which do not express TrkB‐T1 but express Willin, to additionally establish the BDNF–TrkB‐T1–Willin pathway via ectopic expression of TrkB‐T1‐EGFP. We demonstrated that BDNF increased the migration distance of pseudopods, which was not observed in the BSA protein control. Knocking down the expression of Willin using si‐Willin‐2095 abrogated the migration‐promoting effect. The fact that the ectopically expressed TrkB‐T1 in 293 T cells was localized in the cell membrane suggested that BDNF–TrkB‐T1 recruited Willin via the intracellular domain of TrkB‐T1 to promote the migration of pseudopods. Importantly, we confirmed this promotion effect of BDNF in aged CMECs and verified that knockdown of Willin using si‐Willin‐751 in old CMECs abrogates the effect of BDNF in promoting the migration of pseudopods. Furthermore, under the same dose of BDNF treatment (160 ng/ml), the mean migration distance of old CMECs was similar to that of TrkB‐T1‐transfected 293 T cells (Figure 2Ib and 3 g). In addition, BDNF treatment increased the number of polarized cells and the diameter of stress fibres in aged CMECs. Together, these data suggest that the BDNF–TrkB‐T1–Willin pathway is able to increase the migration distance of pseudopods, the polarity of actin and the polymerization of stress fibres to facilitate the migration potential of aged CMECs. Indeed, using a scratch‐healing model, we confirmed that BDNF treatment promoted the migration of aged CMECs.

Willin/FRMD6 was first identified via a yeast two‐hybrid screen of a rat sciatic nerve library using the neuronal transmembrane protein neurofascin as bait (Gunnmoore et al., 2005). Willin was subsequently reported as the human homologue of the Drosophila protein Ex (Expanded) and was given the HUGO nomenclature gene name FRMD6 (Charles et al., 2002). Willin is expressed in the brain, heart, lung, liver and prostate (https://www.genecards.org/cgi-bin/carddisp.p1?gene=FRMD6) and in peripheral nerves, epithelial layers, skin, the placenta, the uterus and the cervix (Charles et al., 2002; Gunnmoore et al., 2005; Madan et al., 2006). In the present study, we first reported that aged CMECs expressed Willin and that Willin was recruited as a downstream effector of TrkB‐T1 to transduce BDNF signals to promote the migration of aged CMECs. As Willin is an upstream component of the Hippo signalling pathway (Angus et al., 2012; Moleirinho et al., 2013), the downstream activation of the Hippo pathway via BDNF–TrkB‐T1–Willin was observed. We confirmed that BDNF treatment for aged CMECs upregulated *MST1*,* MST2*,* LATS1*,* LATS2, Yap and Willin*. In addition, when *Willin* was knocked down in aged CMECs, *MST1*,* MST2*,* LATS1*,* LATS2* and *Yap* genes were upregulated compared with non‐Willin knockdown, whereas in the Willin‐knockdown cells, the expression levels of the *MST1*,* MST2*,* LATS1*,* LATS2* and *Yap* genes upon BDNF treatment were higher than in the non‐BDNF‐treated cells. Importantly, our results also show that BDNF increased the expression of Willin protein and promoted the phosphorylation of MST1/2 and LATS1/2 in old CMECs. These results suggest that an interaction relationship exists between the key effectors of the BDNF, Willin and Hippo pathways (MST1, MST2, LATS1, LATS2 and Yap). Furthermore, Willin was an upstream effector of MST1, MST2, LATS1, LATS2 and Yap, and a negative‐feedback relationship existed to regulate the interactions between Willin and these Hippo pathway key effectors in vivo. As the downstream effectors of Willin, the expression levels of *MST1*,* MST2*,* LATS1*,* LATS2* and *Yap* sensed the downregulation of Willin to upregulate their own expression levels as a feedback response to maintain the pathway activity in vivo.

The dephosphorylation of Yap and its translocation into the nucleus are hallmarks of activation of the Hippo pathway (Zeng & Hong, 2008). Indeed, the western blot results of the nuclear and cytoplasmic fractions revealed that BDNF treatment decreased the level of phosphorylation of Yap in whole‐cell lysates of old CMECs 5 min to 60 min after BDNF treatment. However, knockdown of Willin by si‐Willin‐751 delayed the decrease in Yap phosphorylation. The phosphorylation level of Yap in si‐Willin‐751‐treated old CMECs was maintained at a higher level 15 min after BDNF treatment compared with same time point of BDNF‐treated old CMECs. This suggests that the BDNF–TrkB‐T1–Willin pathway is able to promote the dephosphorylation of Yap in old CMECs. In parallel, the level of phosphorylated Yap in the cytoplasm decreased from 5 min to 60 min after BDNF treatment. However, when Willin was knocked down in old CMECs, BDNF treatment induced a progressive increase in phosphorylated Yap in the cytoplasm, while the level of dephosphorylated Yap in the nucleus was increased from 5 min to 60 min after BDNF treatment. Moreover, when Willin was knocked down in old CMECs, BDNF treatment for 60 min induced a decrease in dephosphorylated Yap in the nucleus. All these results indicate that, in old CMECs, BDNF treatment for approximately 60 min induced dephosphorylation of Yap in the cytoplasm and increased Yap levels in the nucleus, while knocking down Willin in old CMECs abrogated the BDNF‐mediated dephosphorylation of Yap in the cytoplasm and the increased dephosphorylated Yap in the nucleus. Yap immunofluorescence staining also confirmed that BDNF treatment induced translocation of Yap into the nucleus via the BDNF–TrkB‐T1–Willin pathway.

We previously reported that the BDNF–TrkB pathway is able to promote the migration of young CMECs via activation of PI3K/Akt. This effect is mainly attributed to the activation of TrkB‐FL receptor as BDNF treatment significantly increases the expression of TrkB‐FL but not TrkB‐T1, though both receptor isoforms are expressed in young CMECs (Cao et al., 2012). In the present study, we further demonstrate that aged CMECs are able to activate the BDNF–TrkB‐T1–Willin–Hippo pathway to promote their migration. In addition, both young and old CMECs express Willin and key effectors of the Hippo pathway even at similar levels. These results might suggest that the BDNF–TrkB‐T1–Willin–Hippo pathway is not a unique mechanism for old CMECs but also works in young CMECs. With aging, CMECs only expressing TrkB‐T1 might be a key reason why old CMECs activate TrkB‐T1 to recruit Willin to activate the Hippo pathway to transduce the BDNF signal to promote their migration. However, the design and results of the present study cannot answer the question whether BDNF binds equally to both TrkB‐FL and TrkB‐T1 receptors in young CMECs to transduce the BDNF signal via the PI3K/Akt and Willin–Hippo pathways, respectively, or there is a priority for BDNF to activate one or the other, as young CMECs express both TrkB‐FL and TrkB‐T1, which have identical extracellular domain. All these intriguing issues need to be deeply investigated in future studies.

**Figure 6:**
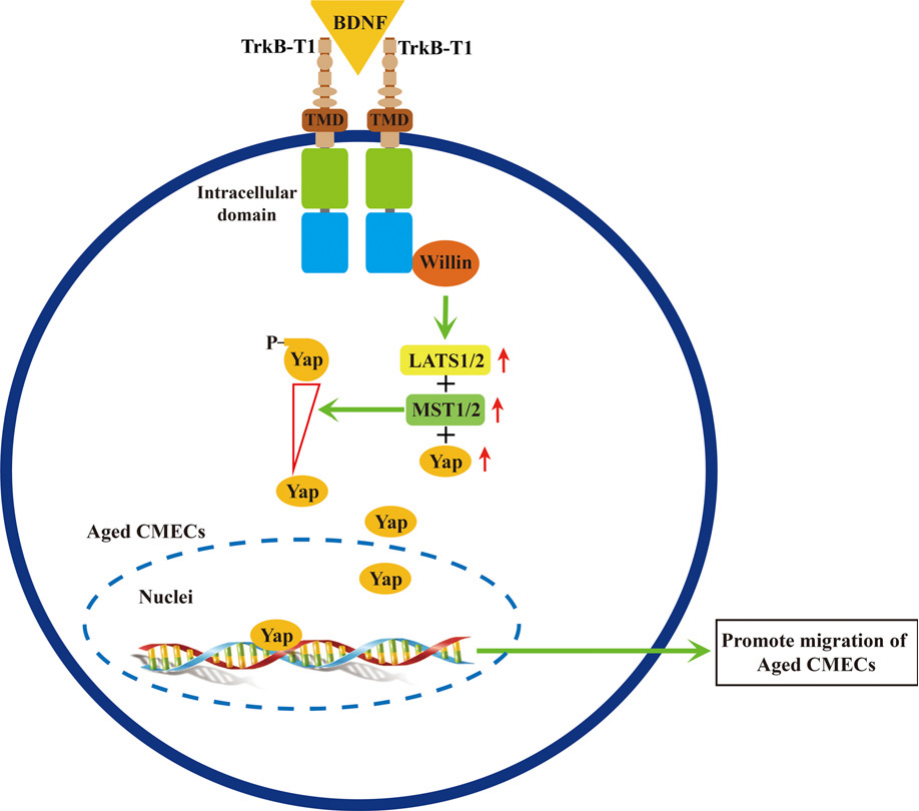
The proposed novel mechanism of the migration of old CMECs via the BDNF–TrkB‐T1–Willin–Hippo pathway. Aged CMECs utilize the TrkB‐T1 receptor to recruit Willin as a downstream effector to transduce BDNF signals. This further upregulates and activates key elements (MST1, MST2, LATS1, LATS2 and Yap) of the Hippo pathway and promotes the translocation dephosphorylated Yap into the nucleus, thus activating the Hippo pathway and promoting the migration of old CMECs. Blue rectangle: the 11‐C‐terminal‐amino acid domain

The findings of the present study suggest a novel mechanism of the BDNF–TrkB‐T1 pathway in aged CMECs. Thus, aged CMECs utilize the TrkB‐T1 receptor to transduce BDNF signals. The BDNF–TrkB‐T1 pathway recruits Willin as a downstream effector to further upregulate key elements of the Hippo pathway. This promotes the dephosphorylation of Yap and facilitates the translocation of dephosphorylated Yap into the nucleus, which activates the Hippo pathway to promote the migration of old CMECs (Figure 6). To our knowledge, this is the first report of the activation of the BDNF–TrkB‐T1–Willin–Hippo pathway in old CMECs, which promotes the migration potential and the migration of aged CMECs. The aging of CMECs leads to changes in the expression of receptor Trk isoforms: among the three isoforms (TrkB‐FL, TrkB‐T1 and TrkB‐T2), only one of its truncated isoforms, TrkB‐T1, continues to be expressed in aged CMECs. The BDNF–TrkB‐T1–Willin–Hippo pathway then promotes the migration of aged CMECs. This aging phenotype might be involved in age‐related dysfunction of the angiogenic potential and other cardiac pathophysiology situations seen in aged hearts. Indeed, recent studies revealed that cardiomyocytes express both TrkB full‐length (TrkB‐FL) and TrkB‐T1 signalling, and both are likely required for the heart to fully contract and relax (Feng et al., 2015; Fulgenzi et al., 2015). However, the expression of TrkB‐T1 is significantly upregulated in failing heart (Feng et al., 2015). Thus, our study in aged CMECs appears to concur with the findings of Feng et al. (obtained in failing cardiomyocytes), suggesting that the BDNF–TrkB‐T1 pathway may be involved in some pathophysiological features of the aging heart. We speculate that the BDNF–TrkB‐T1–Willin–Hippo pathway should be considered a novel target for improving the age‐related decrease in the angiogenic potential in aged hearts to tailor novel strategies to improve the healing and regeneration of aged ischaemic myocardium.

## 4 EXPERIMENTAL PROCEDURES

A detailed method is available in Supporting Information Data S1.

### 4.1 Animals

For details, see Supporting Information Data S1.

### 4.2 Isolation and culture of rat CMECs

For details, see Supporting Information Data S1.

### 4.3 Yeast two‐hybrid screening

For details, see Supporting Information Data S1.

### 4.4 Validation of novel TrkB‐T1 interactor by Co‐IP/WB

For details, see Supporting Information Data S1.

### 4.5 Bimolecular fluorescence complementation

For details, see Supporting Information Data S1.

### 4.6 Transfection of 293T cells with TrkB‐T1‐EGFP

For details, see Supporting Information Data S1.

### 4.7 Time‐lapse analysis for pseudopod

For details, see Supporting Information Data S1.

### 4.8 Semiquantitative analysis of the activity for pseudopod

For details, see Supporting Information Data S1.

### 4.9 In vitro wound healing assay

For details, see Supporting Information Data S1.

### 4.10 Quantitative real‐time PCR

For details, see Supporting Information Data S1.

### 4.11 Nuclear/cytoplasmic fractionation

For details, see Supporting Information Data S1.

### 4.12 Western blotting

For details, see Supporting Information Data S1.

### 4.13 Histochemistry staining for F‐actin

For details, see Supporting Information Data S1.

### 4.14 Immunofluorescent staining for Yap protein

For details, see Supporting Information Data S1.

### 4.15 Statistics

For details, see Supporting Information Data S1.

## AUTHOR CONTRIBUTIONS

Z.W. and Y.C. performed most of the experiments and analysed the data; X.C. and G.X. finished the yeast two‐hybrid screening; Z.Y., H.Z. and X.Q. contributed to the discussion; X.Z., X.S. Y.C., L.L. and N.Z. performed animal preparation and data collection; and D.C. conceived and designed this work and wrote the manuscript.
