# Supplementary figures and images for: The TrkB‐T1 receptor mediates BDNF‐induced migration of aged cardiac microvascular endothelial cells by recruiting Willin
Source: Aging Cell. 2019 Jan 22;18(2):e12881. doi: 10.1111/acel.12881 (PMC6413668; doi:10.1111/acel.12881)

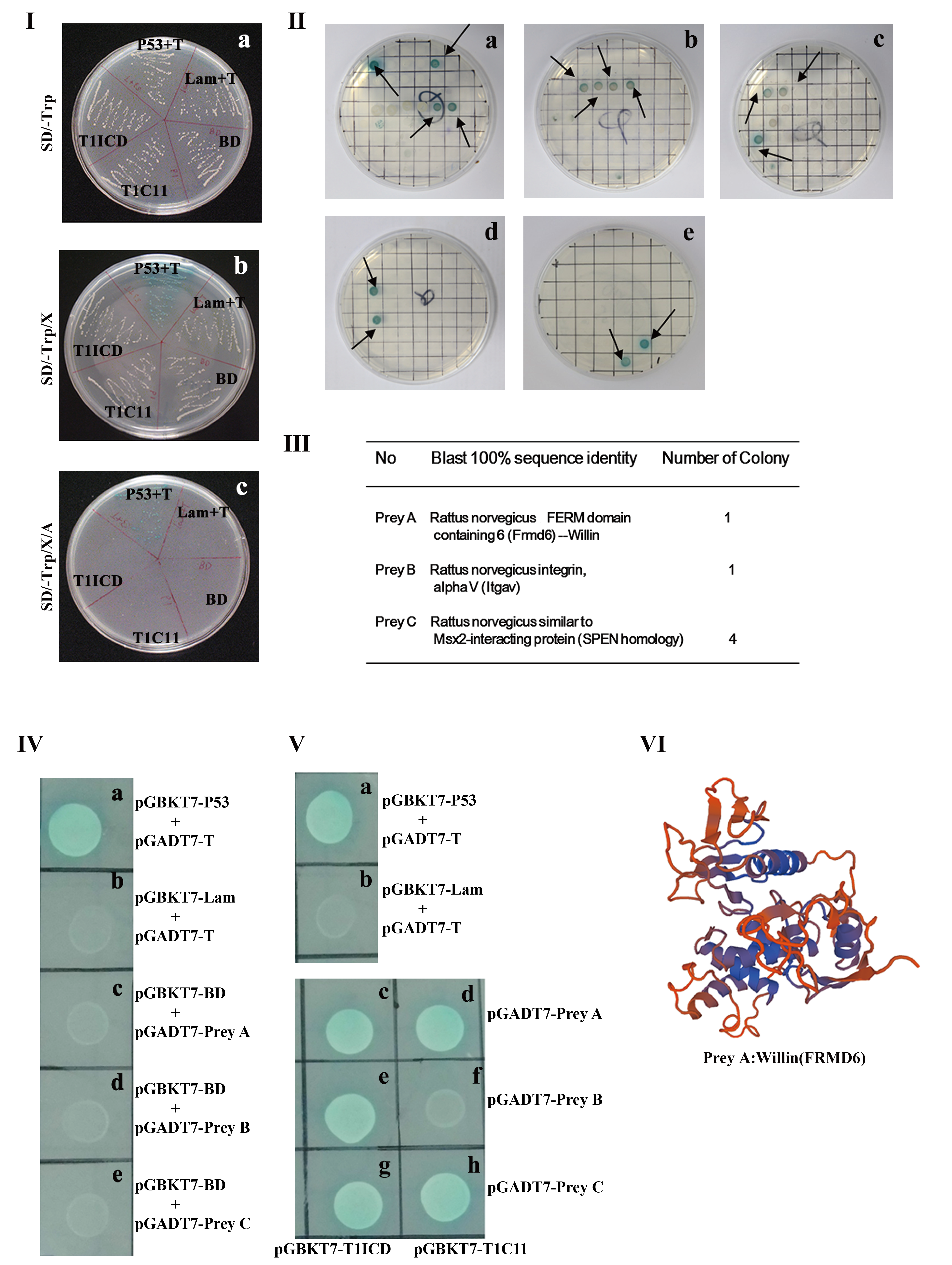

Supplement: Supplementary file 1 [file ACEL-18-e12881-s001.tif]

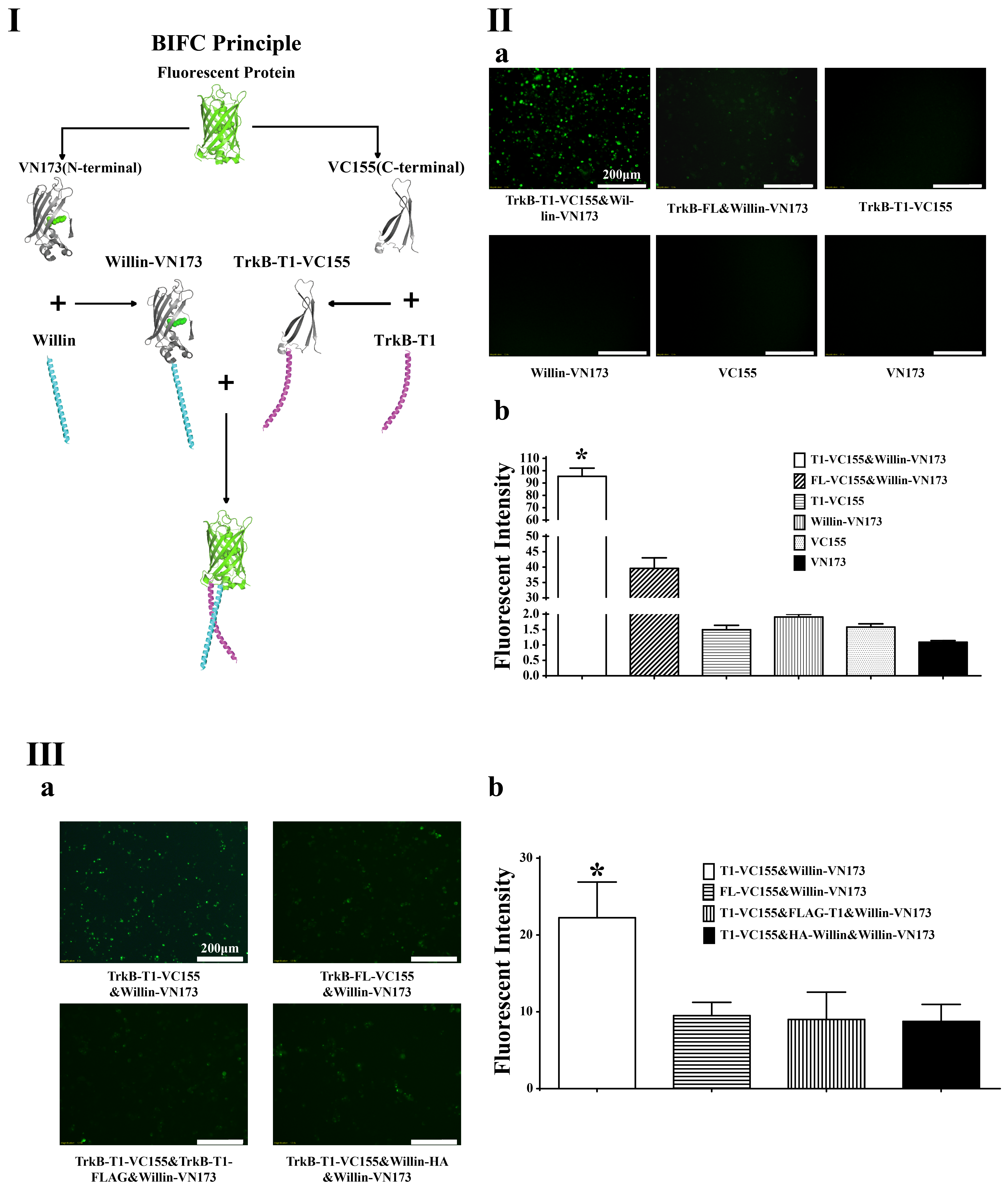

Supplement: Supplementary file 2 [file ACEL-18-e12881-s002.tif]

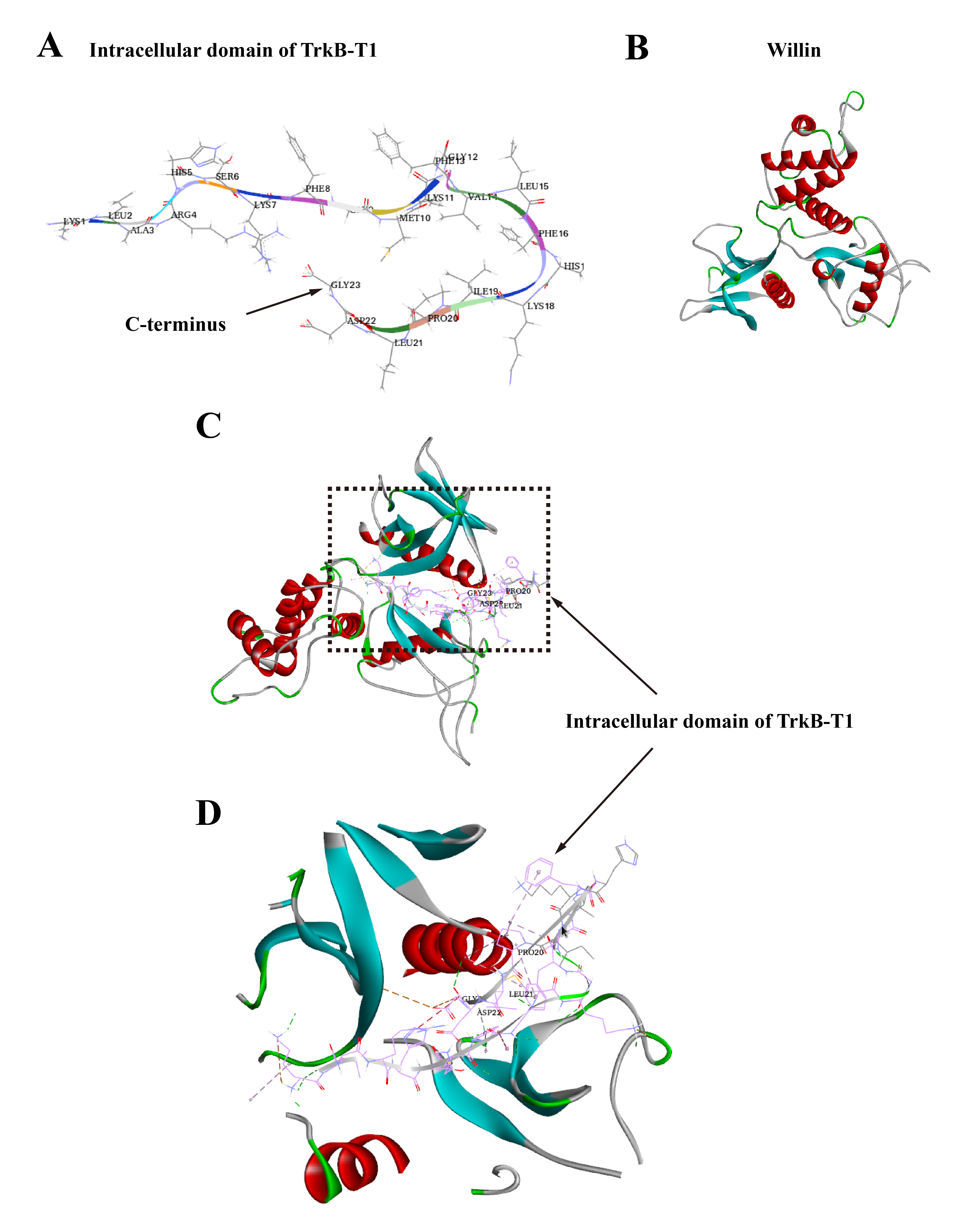

Supplement: Supplementary file 3 [file ACEL-18-e12881-s003.tif]

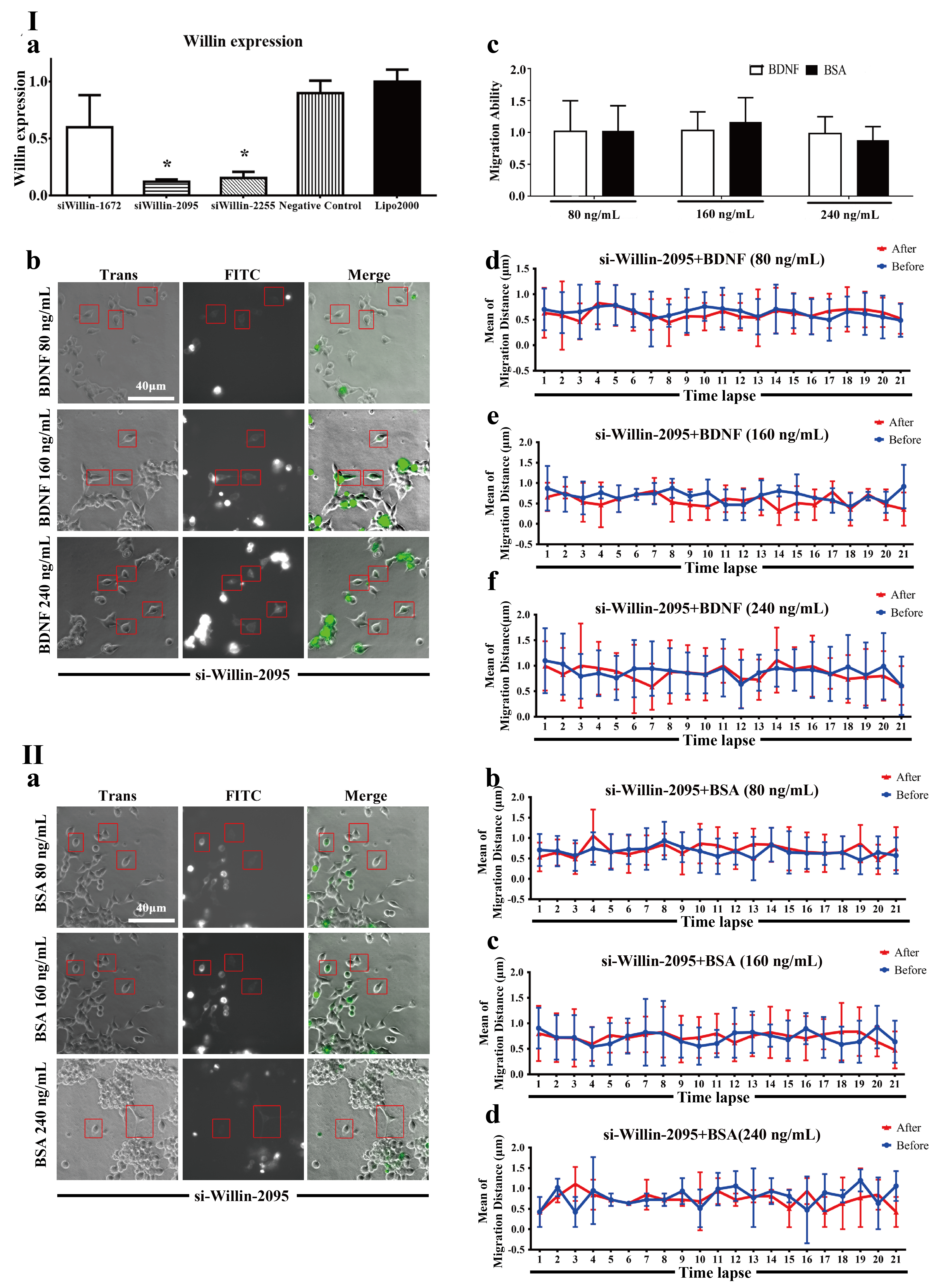

Supplement: Supplementary file 4 [file ACEL-18-e12881-s004.tif]

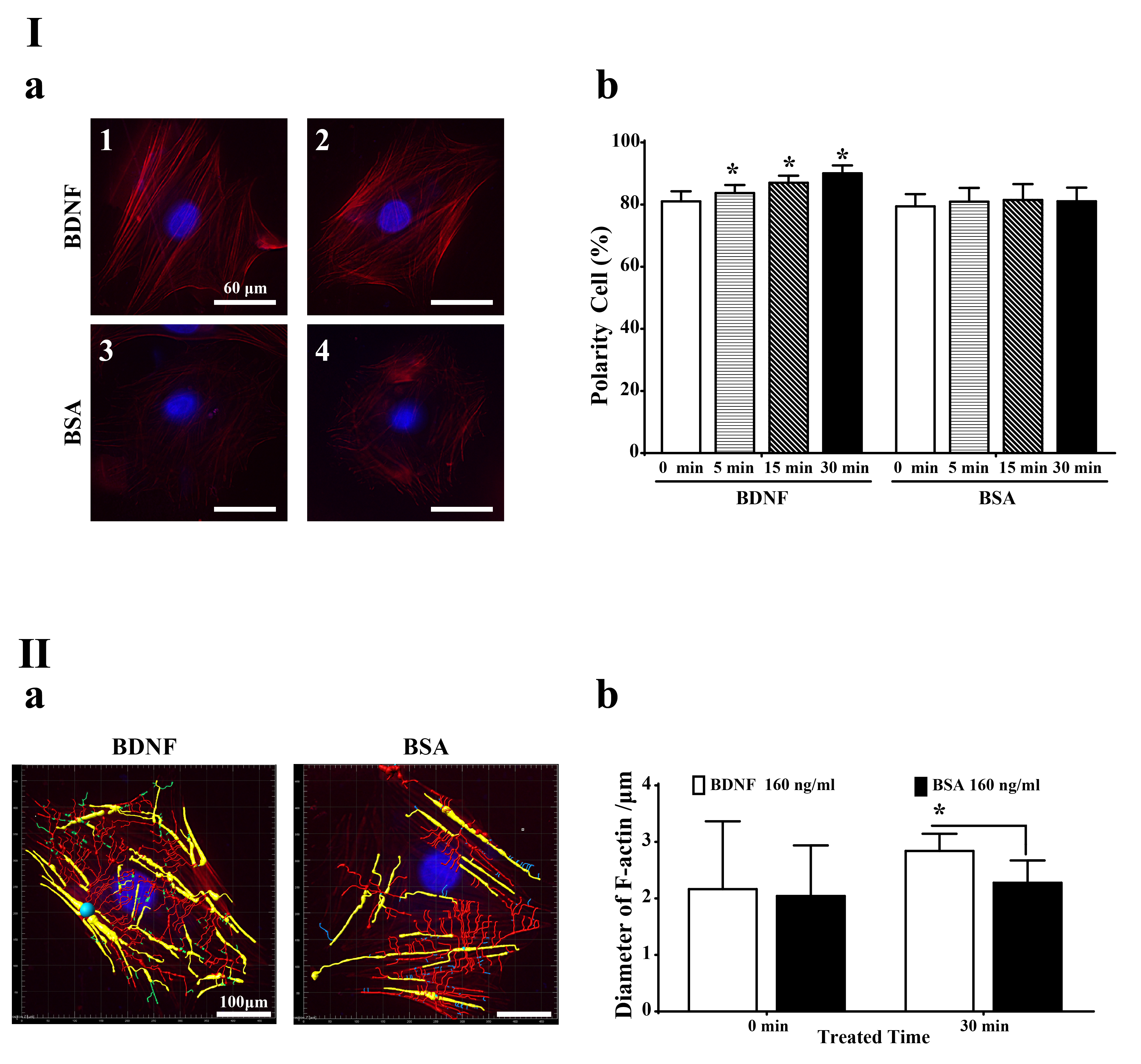

Supplement: Supplementary file 5 [file ACEL-18-e12881-s005.tif]

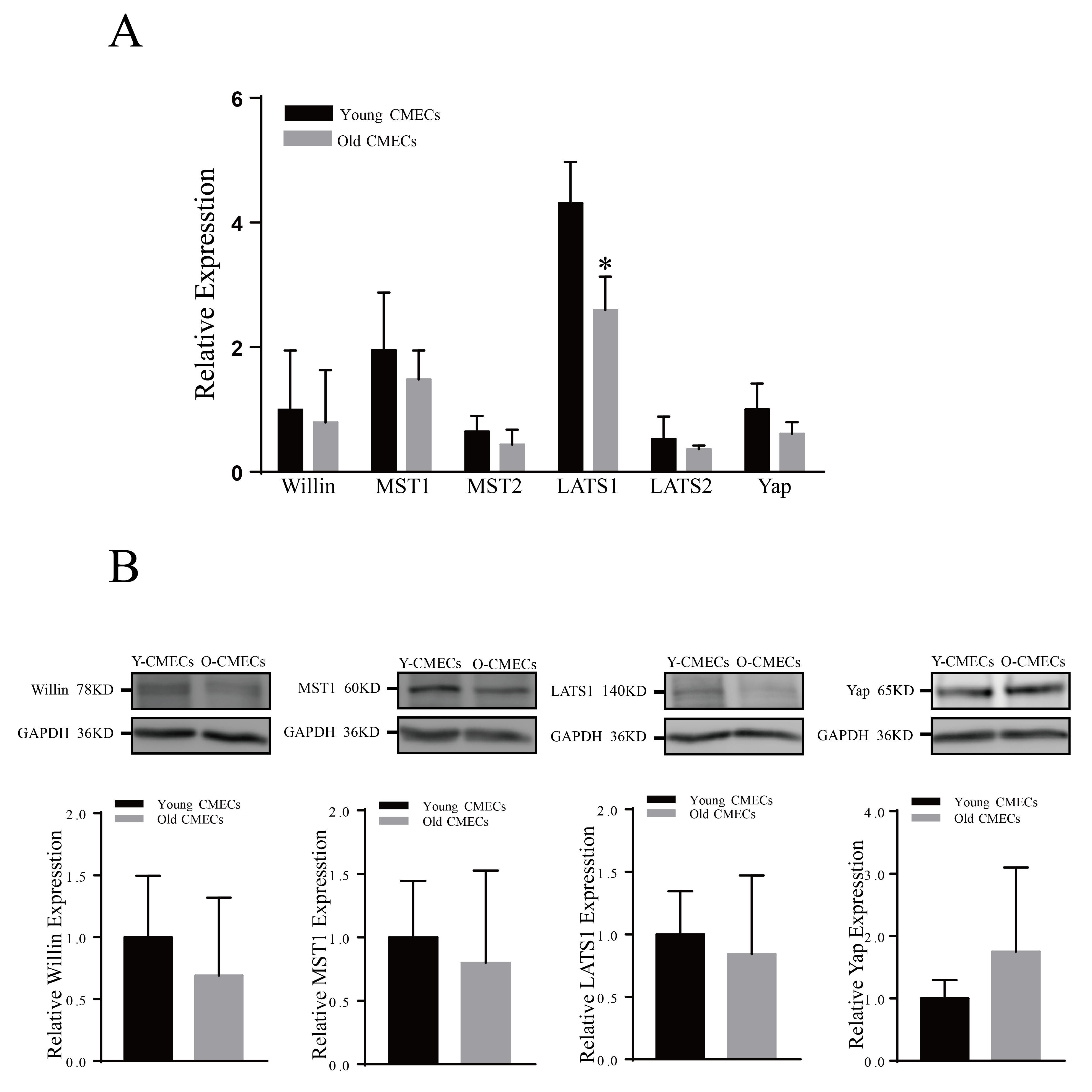

Supplement: Supplementary file 6 [file ACEL-18-e12881-s006.tif]

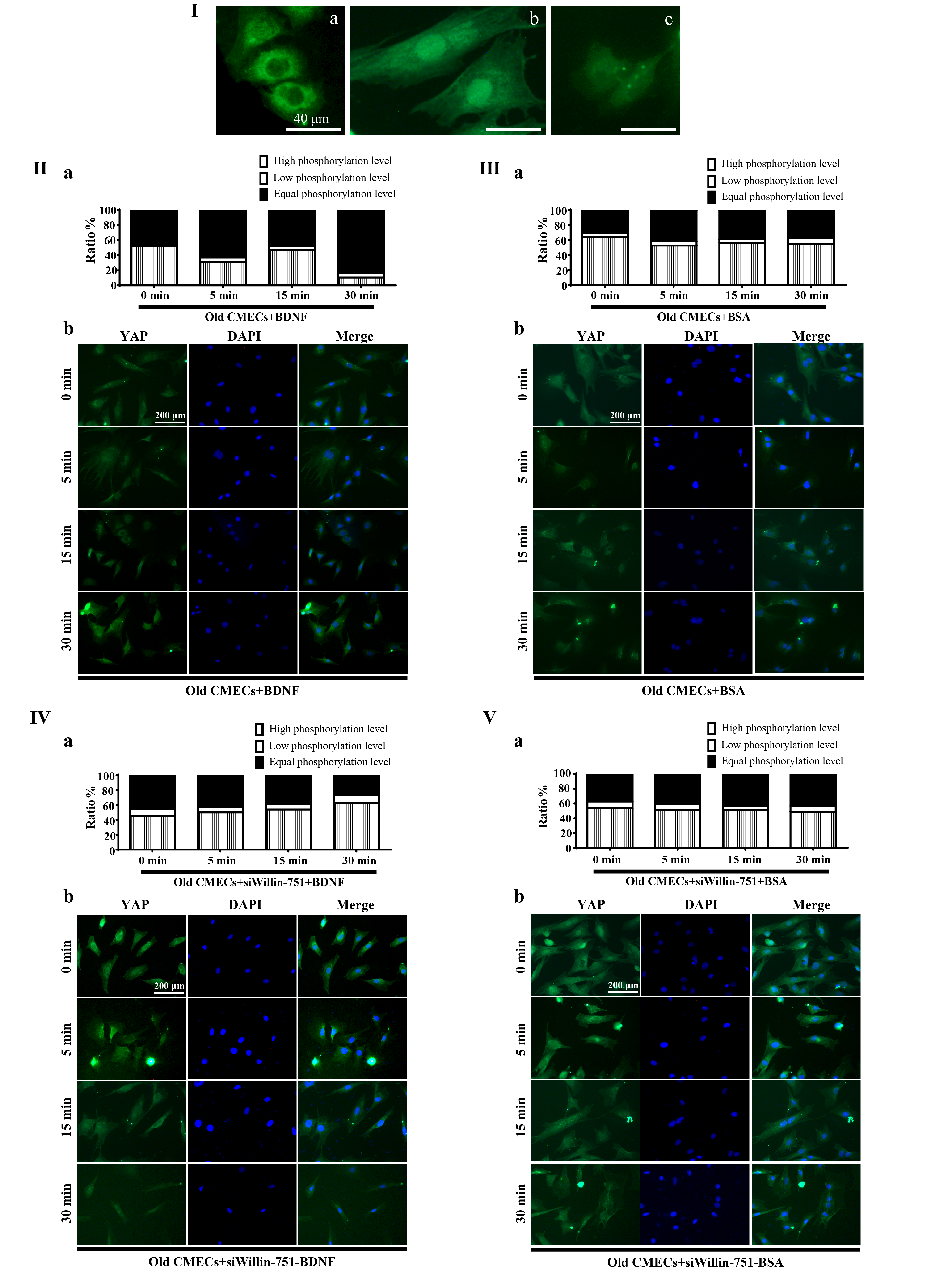

Supplement: Supplementary file 7 [file ACEL-18-e12881-s007.tif]
