# Supplementary material for: The TrkB‐T1 receptor mediates BDNF‐induced migration of aged cardiac microvascular endothelial cells by recruiting Willin
Source: Aging Cell. 2019 Jan 22;18(2):e12881. doi: 10.1111/acel.12881 (PMC6413668; doi:10.1111/acel.12881)
Supplement: Supplementary file 8 [file ACEL-18-e12881-s008.docx]

**Online Supplemental Methods and Data**

**The TrkB-T1 Receptor Mediates BDNF-Induced Migration of Aged Cardiac Microvascular Endothelial Cells by Recruiting Willin**

Zhefeng Wang^1,2,3,4^, Yilin Chen^1,2,3,4^, Xuwei Chen^1,2,3,4^, Xin Zheng^1,2,3,4^, Ganlin Xu^1,2,3,4^, Ziqiang Yuan^5^, Hui Zhao^6^, Wensheng Chen^1,2,3,4^, Lilin Li^1,2,3,4^, Nianjue Zheng^1,2,3,4^, Xiaotao Shen^1,2,3,4^, Yanmei Li^1,2,3,4^, Xufeng Qi^1,2,3,4*^ , Dongqing Cai^1,2,3,4*^

**Running Title:** TrkB-T1 applies Willin inducing Old CMEC Migration

^1^Key Laboratory of Regenerative Medicine, Ministry of Education, Jinan University, Guangzhou 510632, China;

^2^Joint Laboratory for Regenerative Medicine, Chinese University of Hong Kong-Jinan University, Guangzhou 510632, China;

^3^International Base of Collaboration for Science and Technology (JNU), The Ministry of Science and Technology & Guangdong Province, Guangzhou 510632, China;

^4^Department of Developmental & Regenerative Biology, Jinan University Guangzhou 510632, China;

^5^Cancer Institute of New Jersey, Department of Medical Oncology, Robert Wood Johnson of Medical School, USA

^6^Stem cell and Regeneration TRP, School of Biomedical Sciences, Chinese University of Hong Kong, Hong Kong

*Address for correspondence:

Dongqing Cai E-mail: tdongbme@jnu.edu.cn

Xufeng Qi E-mail: qixufeng@jnu.edu.cn

Key Laboratory of Regenerative Medicine

Ministry of Education

Jinan University

Guangzhou, 510632，P.R. China

**ABBREVIATIONS:** CMECs, cardiac microvascular endothelial cell; BDNF, brain Derived neurotrophic factor; TrkB, tyrosine receptor kinase B; MI, myocardial Infarction; VEGF, vascular endothelial growth factor; Co-IP, co-immunoprecipitation; BiFC, bimolecular fluorescence complementation; Yap, Yes-associated protein

**METHODS**

***Animals***

In this study, we used 3-4 month-old (young) and 24-month-old female Sprague-Dawley rats. Animal care, surgery and handling procedures in this study were performed in accordance with the rules of The Ministry of Science and Technology of the People’s Republic of China ([2006]398) and were approved by the Jinan University Animal Care Committee.

***Isolation and culture of rat CMECs***

Young (3-4 months) and Old (24 months) female Sprague-Dawley rats were used. The hearts were removed, and CMECs were isolated and cultured as described in our previous report ([Cai et al., 2003](#_ENREF_1); [Cao et al., 2012](#_ENREF_2)). The culture of the primary isolated cells was defined as Passage 0, and the first subculture was defined as Passage 1. The number of passages was thus defined as the subculture number. All of the CMECs used in this study were less than Passage 10.

***Yeast two-hybrid screening***

We used a Yeastmaker Yeast Transformation System 2 (Clontech, Palo-Alto, CA, USA) to perform yeast two-hybrid screening as described in the instruction manual. Briefly, pGBKT7-T1-ICD vectors (including the full TrkB-T1 intracellular domain) were constructed. Single colony of Y2HGold yeast cells was prepared in YPDA agar plates (Clontech, Palo-Alto, CA, USA) and grown to approximately 0.3 (OD600) at 30°C. The collected Y2HGold yeast cells were finally re-suspended in 600 μL of 1.1×TE/LiAc medium and transformed by mixing with 100 ng of pGBKT7-T1-ICD vectors, 50 μL of competent Y2HGold yeast cells and 5 μL Yeastmaker Carrier DNA plus 500 μL PEG/LiAc medium. After incubating at 30°C for 30 min, 20 μL DMSO was added, and the mixtures were incubated at 42°C for 15 min. The cells were collected by centrifugation, re-suspended in 1 mL YPDA Plus medium (Clontech, Palo-Alto, CA, USA) and then incubated at 30°C for 45 min. The single colony was screened using SD selection medium (as described in the product manual) at 30°C. The SD/-Trp-, SD/-Trp/+X-α-GAL- and SD/-Trp/+X-α-GAL/AbA agar plates were applied to exclude the auto-transcriptional activity of pGBKT7-T1-ICD, as described in the kit instructions.

The old-CMEC cDNA library was constructed by using the Mate & Plate™ Library System (Clontech, Palo-Alto, CA, USA). Total RNA in old CMECs (10^6^) was first extracted by using RNeasy Plus Mini Kits (Qiagen, Valencia, CA, USA), and the single-stranded cDNAs (sscDNA) were then synthesized from 2 μg of isolated old-CMEC total RNA using CDSIII/6 primers, as described in the instruction manual. Double-stranded cDNAs (dscDNA) were further acquired by 20 cycles of long-distance PCR using 10× Advantage^®^ 2 PCR buffer (provided by kit) with 2 μL first-strand cDNA according to the manufacturer’s instructions. The purified dscDNA of the old CMEC cDNA Library with the linearized pGADT7-Rec AD cloning vector (Clontech, Palo-Alto, CA, USA) were co-transformed into Y187 yeast competent cells to construct the old-CMEC cDNA yeast two-hybrid library (1.75125×10^7^ cfu, >1×10^7^ cfu, titer: 1.548×10^8^ cfu/mL). The intracellular domain of tropomyosin receptor kinase B truncated isoform type 1 (TrkB-T1-ICD) was designed as a bait to identify potential candidate interacting proteins from the old-CMEC cDNA library expressed in the Y187 yeast. The 23-amino acid encoding sequence for trkB-T1-ICD was amplified by re-annealing the pGBKT7-T1-ICD primer with the following sequences: Sense: 5’-AATTCAAGTTGGCGAGACATTCCAAGTTTGGCATGAAAGGTTTTGTTTTGTTTCATAAGATCCCCCTGGATGGGTAGG-3’; Antisense: 5’-GATCCCTACCCATCCAGGGGGATCTTATGAAACAAAACAAAACCTTTCATGCCAAACTTGGAATGTCTCGCCAACTTG-3’.

The product was recovered by 3% agarose gel, and the sequence was confirmed by DNA sequencing. The collected trkB-T1-ICD fragment was inserted into the Y2H yeast GAL4 binding domain vector pGBKT7 (Clontech, Palo-Alto, CA, USA) using the EcoRI-BamHI sites to generate the BD-bait plasmid (pGBKT7-T1-ICD). Subsequently, the pGBKT7-T1-ICD was transformed into the Y2HGold yeast strain, and the positive colonies were selected on SD/-Trp medium.

After the correct expression of trkB-T1-ICD was confirmed by Western blot, the constructed Y187 yeasts that included the old-CMEC cDNA library were screened using the Y2HGold yeast-expressed bait of pGBKT7-T1-ICD via yeast-mating. The mated clones were selected on SD medium fortified with both tryptophan and leucine to ensure successful mating. Finally, the interacting partners were screened on SD/-Leu/-Trp/-His/-Ade/+X-α-Gal/AbA agar plate. A direct Y2H assay was applied to confirm the reliability of the constructed yeast two-hybrid system. The pGBKT7-BD (control) and pGBKT7-T1-ICD were transformed into Y2HGold to exclude possible false positives of the vector transformation. The screened positive Y2H yeast colon was selected and grown in YPDA medium at 30°C for 16-24 h. An EZgene^TM^ yeast plasmid kit (Biomiga, San Diego, CA, USA) was used for yeast plasmid isolation according to the manufacturer’s instructions. The collected plasmids were dissolved by RNase-free water, and DNA sequencing was performed to identify the sequence of the selected positive colony.

***Validation of novel TrkB-T1 interactor by Co-Immunoprecipitation /*** ***Western blot***

Our yeast two-hybrid screening showed that Willin/FRMD6 was a possible candidate that interacted with TrkB-T1 in old CMECs. Therefore, 3×HA-tagged Willin (pIRES2-3×HA-Willin, subcloned into the XhoI and BamHI site of pIRES2-3’-3×HA) and Flag-tagged TrkB-T1 (pFLAG-TrkB-T1, subcloned into the BamHI and EcoRI site of pFXF–3’-FLAG) were applied for Co-Immunoprecipitation (Co-IP)/ Western blot (WB). Human embryonic kidney HEK 293T cells (293T cells) were grown in 35-mm plates with 3 mL Dulbecco's modified Eagle's medium (DMEM) with 10% serum, and HA-tagged Willin plasmids and Flag-tagged TrkB-T1 plasmids were transfected with HB-TRLF-1000 (LipoFiter^TM^, Hanbio, Shanghai, China). The transfected cells were harvested with 0.25% trypsin-EDTA at 37°C after 36 h. The mixture was centrifuged at 1000 ×*g* for 5 minutes at 4°C. After discarding the supernatant, the pellet was resuspended in 300 μL ice-cold IP lysis/wash buffer (50 mM Tris, 150 mM NaCl, 0.1%-0.5% TritonX-100, pH 7.5) with 1% protease inhibitors (1:100, Halt Protease Inhibitor Cocktail, Thermo, Waltham, MA, USA). The lysate was incubated on ice for 5 minutes under periodic mixing and centrifuged at 13,000 ×*g* for 10 minutes at 4°C to pellet the insoluble material. Cells only transfected with HA-tagged Willin or FLAG-tagged TrkB-T1 were used as controls. Next, the protein A/G magnetic beads (Bimake, China) mixture (50 μL) was conjugated to 20 μg HA antibody (Transgene, USA) 120min. The antibody/magnetic beads mixture and lysate mixture was incubated overnight at 4°C. After magnetic isolated, the mixture was centrifuged at 1000 ×*g* for 1 minute, and the supernatant was removed. The pelleted magnetic beads complex was washed 3 times with PBS and magnetic isolated 30 s. The pellet was resuspended with 50 μL of Elution buffer (0.1 M-0.2 M Glycine, 0.1%-0.5% detergent, pH 2.5-3.1 (or 0.1 M citric acid, 0.1%-0.5% detergent, pH 2.5-3.1 or 2.5% Acetic Acid, 0.1 M -0.2 M Glycine, 0.1%-0.5% detergent). The mixture was incubated at 100°C for 10 minutes and magnetic isolated 30 s to collect the eluate. All the collected eluates were run in SDS-PAGE gel (10% polyacrylamide gel) electrophoresis. Proteins in the gel were transferred to polyvinylidene difluoride (PVDF) membranes (Millipore, Bedford, MA, USA), and anti-FLAG antibody (1:1000, Sigma, St. Louis, MO, USA) was used to detect binding.

***Bimolecular fluorescence complementation (BiFC)***

BiFC is based on the reconstitution of a fluorescent protein complex from two separate non-fluorescent fragments, which is induced by the association of two interacting partners fused to the fragments ([Hu, Chinenov, & Kerppola, 2002](#_ENREF_3); [Kerppola, 2006](#_ENREF_4); [Shyu, Liu, Deng, & Hu, 2006](#_ENREF_5)). Plasmids pBiFC-FLAG-VN173 (Plasmid No.22010, Addgene, Cambridge, MA, USA) and pBiFC-HA-VC155 (Plasmid No.22011, Addgene, Cambridge, MA, USA), coding for the N-terminal (VN: 1-173) and C-terminal (VC: 155-238) fragments of Venus, were applied to construct BiFC fragments. Briefly, the intracellular domains of encoding sequences for rat TrkB-T1 (TrkB-T1: 5’-32 bp to 476 bp-3’) and Willin were amplified by PCR from constructed vectors, which included full-length complimentary DNAs, and cloned into the EcoRI site of pBiFC-HA-VC155 and pBiFC-FLAG-VN173, respectively. Willin and TrkB-T1 were also HA-tagged (pHA-Willin, subcloned into the EcoRI site of pFXG-3’-HA) and FLAG-tagged (pFLAG-TrkB-T1, subcloned into the BamHI-EcoRI site of pFXF-3’-FLAG) as competitors for Willin- and TrkB-T1-competitive BiFC, respectively. Plasmids pBiFC-bJun-VC155 (Plasmid No.27098, Addgene, Cambridge, MA, USA) and pBiFC-bFos-VN173 (Plasmid No.22013, Addgene, Cambridge, MA, USA) were used as positive controls. bJun interacts with bFos, which induces binding of the downstream VC155-domain of green fluorescent protein and the VN173-domain of green fluorescent protein for full green fluorescent protein. The pBiFC-bJun-VC155 plasmids (Plasmid No.22012, Addgene, Cambridge, MA, USA) and mutated pBiFC-ΔbFos-VN173 plasmids (Plasmid No.22014, Addgene, Cambridge, MA, USA, carrying a small deletion in the zip region to eliminate specific dimerization with bJun) were used as negative controls. All constructs were sequenced for verification before use. The constructs were transformed with LipoFiter^TM^ (Hanbio, Shanghai, China) and co-expressed in 293T cells. T1-VC155/Willin-VN173 construct was used as the experimental group. The BiFC results were captured using a fluorescence microscope (CKX41; Olympus, Tokyo, Japan) and analyzed according to the ratio of the intensity of fluorescence between the experimental and control groups. For competitive BiFC, IPP image software (Image-Pro Plus 6.0) was applied to analyze the integral optical density (IOD), and the mean of fluorescent intensity was applied for comparisons among groups. Three replicates of each of specimen were performed.

***Bioinformatic prediction of potential critical amino acids for binding between TrkB-T1 and Willin***

For the sequence alignment, the rat protein sequences for TrkB-T1 and Willin were retrieved from NCBI database. The protein crystal structures of TrkB-T1 and Willin were blasted by Discovery studio 4.5 (Accelrys Software Inc., San Diego, CA, USA). For the TrkB-T1, as no any homologous crystal structure was identified, therefore, the whole sequence of intracellular domain of TrkB-T1 was applied to construct the linear model first by Discovery studio 4.5, and then the 3D model of TrkB-T1 was constructed by Gromacs program (Version4.6.7) (RMSD<3nm; RMS fluctuation<0.08nm). The Ramachandran plot analysis (residues in allowed region 17 94.4%; residues in marginal region 1 5.6%; residues in disallowed region 0 0%) and Profile-3D analysis (verify score: 3.23; verify expected high score: 10.25; verify expected low score: 4.61) both showed that the constructed 3D model of TrkB-T1 fits the quality for homology modeling. For the Willin, the FERM domain protein crystal structure of Ezrin was screened as homologus model due to its top listed identity (sequence identity=22.8% and sequence similarity=48.6%). Therefore, the 9-322 amino acids of Willin included in FERM domain was applied for homology modeling by Discovery studio 4.5. The Ramachandran plot analysis (residues in allowed region 265 93.3%; residues in marginal region 14 4.9%; residues in disallowed region 8 1.8%) and Profile-3D analysis (verify score: 100.31; verify expected high score: 142.93; verify expected low score: 64.32) both showed that the constructed 3D model of Willin fits the quality for homology modeling. ZDOCK and RDOCK were applied for prediction of interaction of amino acids between TrkB-T1 and Willin. The predicted potential critical amino acids for binding between TrkB-T1 and Willin were decided by qualified values of interface nonbond interaction and hydrogen bond.

***Transfection of 293T cells with TrkB-T1-EGFP***

To establish the ectopic expression of TrkB-T1, the constructed pTrkB-T1-EGFP vector (including the full encoding sequence of TrkB-T1 and fused downstream with EGFP) was transfected into 293T cells using LipoFiter^TM^ (LipoFiter^TM^, Hanbio, Shanghai, China). The detailed procedure was followed according to the manufacturer’s instructions. Briefly, 293T cells were cultured to approximately 70% confluence in 24-well plates. The pTrkB-T1-EGFP vector (0.8 μg) was mixed with 50 μL DMEM (pH=7.4), while the LipoFiter^TM^ reagent (2.4 μL) was mixed with 50 μL DMEM (pH=7.4). The prepared vector mixture and LipoFiter^TM^ mixture were first mixed and incubated for 20 min at room temperature. Then, the mixture was added to prepared 293T cells and cultured for 24 h at 37°C in 5% CO_2_ and 95% air. The pEGFP vector (0.8 μg) was applied as a control.

***Time-lapse Analysis for pseudopod***

For the analysis of CMECs, old CMECs were digested with 0.25% trypsin-EDTA at 37°C at approximately 80% confluence. After washing three times with PBS (pH=7.4), the cell pellets were resuspended with DMEM. A total of 1×10^4^ cells was added to a 24-well glass bottom plate and cultured with DMEM containing 10% FBS for 6 hours at 37°C in 5% CO_2_ and 95% air. After the cells attached to the plate, the medium was changed to serum-free DMEM overnight, and a time-lapse analysis of the cells was conducted for the following 16 hours. Three replicates of each of the specimens were examined. The results of the present study are from 3 old rats. For the analysis of 293T cells, a total of 1×10^4^ cells was added to a 24-well glass bottom plate and cultured with DMEM containing 10% FBS for 6 hours at 37°C in 5% CO_2_ and 95% air. After the cells attached to the plate, the medium was changed to serum-free DMEM overnight, and a time-lapse analysis of the cells was conducted for the following 16 hours. Three replicates of each of the specimens were examined.

To evaluate the knockdown effects of Willin, the siRNAs for human Willin (si-Willin-1672, sense: GCAUUGAGGCAGACACCAATT, antisense: UUGGUGUCUGCCUCAAUGCTT; si-Willin-2255, sense: GCAGUGCAUCAACAUCCAATT, antisense: UUGGAUGUUGAUGCACUGCTT and si-Willin-2095. sense: CCACUGAUCGACACAGCUUTT, antisense: AAGCUGUGUCGAUCAGUGGTT; GenePharma; China) and rat Willin (si-Willin-550, sense: GCAUUGAGGCAGACACCAATT, antisense: UUGGUGUCUGCCUCAAUGCTT; si-Willin-751, sense: GCAGUGCAUCAACAUCCAATT, antisense: UUGGAUGUUGAUGCACUGCTT and si-Willin-1059, sense: CCACUGAUCGACACAGCUUTT, antisense: AAGCUGUGUCGAUCAGUGGTT, GenePharma, China) were transfected into the TrkB-T1 transfected-293T cells (green fluorescent positive) and old CMECs using Lipofectamine® RNAiMAX Reagent (Life Technologies, Foster City, CA, USA), respectively. As si-Willin-2095 and si-Willin-751 induced the best knockdown effects of Willin expression in human 293T cells and old rat CMECs, si-Willin-2095 and si-Willin-751 were selected for the Willin knockdown experiments in the present study. In the present study, the si-Willin-2095- and si-Willin-751-transfected 293T cells and old CMECs were referred to as si-Willin-2095-293T cells and si-Willin-751-old CMECs.

The TrkB-T1-transfected 293T cells, si-Willin-2095-293T cells, old CMECs and si-Willin-751-old CMECs were imaged in DMEM supplemented with 30 mM HEPES-NaOH (pH=7.4) using in an Olympus (IX81; Olympus, Tokyo, Japan) inverted microscope with a 40× PlanFLN objective (NA 0.6), a Q-image CCD camera and a heated stage (maintained at 37°C in 5% CO_2_ and 95% air). Images were captured every 15 seconds for 15 min as control images. Then, BDNF (80, 160 and 240 ng/mL for 293T cells; 160 ng/mL for CMECs) was added to the individual wells for an additional 15 min and imaged as the BDNF treatment groups.

***Semi-quantitative analysis of the activity for*** ***pseudopod***

Image-Pro Plus version 6.3 was used to track and analyze the migration distance of the pseudopod for the time-lapsed cells. The objective lens was defined to first set the spatial calibration. The trajectories of individual pseudopods were targeted using a manual format model to locate of migration tracks from the starting point (t=0 minute). The migrating distance was calculated by the software. The migrating distance was defined as the distance measured between track points in 15-second intervals for each captured frame up to 15 min. Graph Pad Prism 6 software was used to generate line graphs from the recorded images for the BDNF treatments and controls. In this study, 473 individual CMECs from 3 old rats (3 protrusions / each cell) and 144 individual 293T cells (3 protrusions/each cell) were investigated, respectively.

***In vitro Wound Healing Assay***

Old CMECs (10^5^) were plated in each well of a 24-wellculture plate. For the knockdown experiments, young and old CMECs at approximately 60-70% confluence were transfected for two consecutive days with si-Willin-751-old CMECs using Lipofectamine^®^ RNA iMAX reagent (Life Technologies, Foster City, CA, USA) and allowed to grow until reaching 100% confluence. Monolayers of confluent cultures were gently scratched with a sterile micropipette tip, and BDNF (160 ng/mL) was added as the treated group, and same amount of BSA was added as the control. The migration toward the wound was monitored for up to 24 hours. Phase-contrast images were captured after the scratch and BDNF treatment at 0, 16 and 24 hours. The percentage of the scratch covered by cells was measured and calculated as the percentage of the invaded area with respect to the initial wound area using Image-Pro Plus 6.0 Three replicates of each of the specimens were examined. The results of the present study are from three old rats.

***Quantitative real-time PCR***

The expression levels of *Willin, MST1, MST2, LATS1*, *Yap* were quantified using SYBR green-based real-time PCR. The reaction mixture was composed of 10 μL PCR master mix (Toyobo, Osaka, Japan), 0.8 μL primer, 7 μL PCR-grade water and 1 μL cDNA template. The primers used were (5’-3’): for *Will-in*: (sense: human- CTACTCCAGCAGTGCCATCCA, rat-GGAGGACTCGCAAGATGATGAA) and (antisense: human-CTACTCCAGCAGTGCCATCCA, rat-GCTGGAGGTGGAAGAACTGATT); for *MST1*: (sense: human-AAACTTGGAGAAGGGTCCTATGG, rat-CCTCCTCCCACATTCCGTAAGC) and (antisense: human-ATGAGGGCTGTCACATTGCTG, Rat-GAACCATCGTGCCAGAGTCCAT); for *MST2*: (sense: human- CGGAGTTACGGGAAAGTTGGTC, rat-ATTCCTACAAACCCACCACCAACA) and (antisense: human-CAGAGAGAGGGACCTGGTGGAC, rat-GCTCTCGCTGCTGCTCCTCAT); for *LATS1*: (sense: human-CTGAAGCCATTAGAGCGGAGAG, rat-GCCAGACCTATCAATGCCACCAT) and (antisense: human- GTGTTTGTGCCAAGAAAGGAGG, rat-CTTCCTACATCCGCCTGTGAGTTG); for *Yap*: (sense: human-AGCAACTCCAACCAGCAGCA, rat-CAGGAGACACCATCAGCCAGAG) and (antisense: human-TTTGAGTCCCACCATCCTGCT, rat-GCCAACACAGACTCCACATCCA) and for *β-actin*: (sense: human-TCATGA AGTGTGACGTTGACATCCGT, rat-TCATGA AGTGTGACGTTGACATCCGT) and (antisense: human-CCTAGAAGCATTTGCGGTGCACGATG, rat-CCTAGAAGCATTTGCGGTGCACGATG). Amplifications were performed starting at 95°C for 30 s, followed by 40 cycles of denaturation at 95°C for 5 s, combined primer annealing at 60°C for 10 s and then extension at 72°C for 15 s using a **Mini-Opticon System** (Opticon Monitor 3.1, Bio-Rad, Hercules, CA, USA). The cycle threshold (C_T_) values for the individual reactions were determined using Opticon Monitor 3.1 software (Bio-Rad, Hercules, CA, USA). All cDNA samples were amplified in triplicate and were normalized against β-actin on the same plate. The results of this study were from three old rats.

***Nuclear/cytoplasmic fractionation***

A ProteinExt^TM^ Mammalian Nuclear and Cytoplasmic Protein Extraction Kit (TransGen, Beijing, China) was used to extract proteins included in the nuclear/cytoplasmic fractionation. After reaching the desired confluence, the old CMECs (2×10^6^) were harvested with 0.25% trypsin-EDTA at 37°C, pelleted at 1000 ×*g* for 3 min, washed with PBS and then pelleted at 1000 g for 3 min. The pellet was resuspended in 0.1 mL of CPEB-I (provided by kit) and kept on ice for 10 min with periodic mixing. Then, 5.5 μL CPEB-II (provided by kit) was added and kept on ice for 1 min, followed by centrifugation at 16000 ×*g* at 4°C for 5 min. Then, the supernatant (cytoplasmic proteins) was transferred into a new tube for further analysis. The rested pellet was resuspended in 30 μL of ice-cold NPEB, incubated on ice for 30 min with periodic mixing, and centrifuged at 16000 ×*g* at 4°C for 10 min. The supernatant (nuclear proteins) was transferred into a new tube for further analysis. Protein sample buffer was added to both the nuclear and cytoplasmic protein fractions, and then the western blot analysis was performed.

***Western blotting***

Cell lysates produced from old CMECs and si-Willin-751-old CMECs treated with (160 ng) or without BDNF were used in the western blot analysis. In addition, the cell lysates of nuclear and cytoplasmic protein fractions that were produced from old CMECs and si-Willin-751-old CMECs treated with (160 ng/mL) or without BDNF were used for western blotting to analyze the expression levels in the nuclei and cytoplasm. Briefly, the cells were lysed in RIPA (Beyotime, Shanghai, China) with 1 mM PMSF and a protease inhibitor cocktail (Halt Inhibitor Cocktail, Thermo, MA, USA; Halt Phosphatase Inhibitor Cocktail, Thermo, MA, USA). The proteins were denatured in loading buffer (5×SDS-PAGE loading buffer: 0.5 M Tris-HCl, pH 6.8, 0.05% beta-mercaptoethanol, 1% SDS, 0.005% bromophenol blue, 50% glycerol) for 10 min at 95°C, electrophoresed using a 10% SDS-PAGE gel, and then transferred to a PVDF membrane (Bio-Rad, CA, USA). The membranes were incubated overnight with the appropriate primary antibody (Rabbit anti-Yap, 1:1000, CST, Danvers, MA, USA; Rabbit anti-pYap, 1:1000, CST, Danvers, MA, USA; Rabbit anti-MST1, 1:1000, CST, Danvers, MA, USA; Rabbit anti-phospho-MST1(Thr183)/MST2(Thr180), 1:1000, CST, Danvers, MA, USA; Rabbit anti-LATS1, 1:1000, CST, Danvers, MA, USA; Rabbit anti-phospho-LATS1(Ser909), 1:1000 , CST, Danvers, MA, USA; Rabbit anti-phospho-LATS1(Thr1079), 1:1000 , CST, Danvers, MA, USA; Rabbit anti-Willin, 1:1000, CST, Danvers, MA, USA; Mouse anti-GAPDH, 1:5000, Proteintech, Chicago, IL, USA) at 4°C and then with a secondary antibody conjugated to horseradish peroxidase. Immunoreactive bands were detected using an ECL kit (Millipore, Billerica, MA, USA) and were analyzed using GeneSnap (Sygene). In this experiment, three old rats were used to prepare CMECs, and the isolated CMECs from each animal were used individually for the experiments.

***Histochemistry staining for F-actin***

The BDNF (160 ng/mL)-treated old CMECs (0, 5, 15 and 30-min) and non-BDNF- treated old CMECs were fixed for 15 minutes using fresh prepared methanol-free 4% paraformaldehyde and then rinsed three times with PBS (pH=7.4) for 5 minutes each. Phalloidin (1:200, CST, Danvers, MA, USA) was added to the cells and incubated for 30 minutes at room temperature. After rinsing with PBS and sealing with glycerol, the stained cells were captured via fluorescent microscopy (60×; Leica, Germany) at a wavelength of 554 nm. Three old rats were applied to prepare CMECs, and the isolated CMECs from each animal were used individually for the experiments.

***Immunofluorescent staining for Yap protein***

BDNF (160 ng/mL)-treated old CMECs (0, 5, 15 and 30-min) and BSA-treated old CMECs (control) at parallel time-points, which were cultured on polylysine-coated glass, were fixed with freshly prepared 4% paraformaldehyde for 15 min and then rinsed three times with PBS (pH=7.4; 5 min/each). Rabbit anti-Yap antibody (1:100, CST, Danvers, MA, USA) was added and incubated at 4°C overnight. After washing three times with PBS, goat anti-rabbit conjugated with FITC secondary antibodies (1:100, Proteintech, Chicago, IL, USA) was added and incubated for 1 h at room temperature. After rinsing three times with PBS, the stained cells were sealed with glycerol and captured under fluorescent microscopy (40×; Leica, Germany) using the appropriate excitation wavelength. Three old rats were applied to prepare the CMECs, and the isolated CMECs from each animal were used individually for the experiments.

***Statistics***

All measured data are presented as the means ± standard errors. Two-tailed student’s *t*-test was used to calculate the statistical significance between two groups. The one-way (ANOVA) analysis test was used to determine among three or more groups. A *P* value <0.05 was considered statistically significant. All the results of present study represent three independent experiments.

***Data availability***

The authors declare that data supporting the findings of this study are available within the article.

**Supplementary Materials**

**

**

**Figure S1 Yeast two-hybrid screening identifies Willin/FRMD6 as a potential downstream effector of TrkB-T1. I:** The autoactivation activity of the bait proteins in yeast cells, the pGBKT7-T1-ICD and pGBKT7-T1-C11 bait plasmids were transformed into Y2HGold cells, and subsequently the transformants were grown on SD/-Trp, SD/-Trp/X-α-Gal and SD/-Trp/X-α-Gal/AbA. Autoactivation activities from the bait would enable the expression of reporter genes and result in blue colonies on SD/-Trp/X-α-Gal and SD/-Trp/X-α-Gal/AbA plates. The results showed that no autoactivation activity was detected from pGBKT7-T1-ICD and pGBKT7-T1-C11. **II:** Y2HGold cells containing the pGBKT7-T1-ICD plasmid were used to mate with Y187 cells harboring the established old cDNA library of CMECs. Based on the number of colonies on different selection plates, the mating efficiency was calculated to be 6.8%. The fifteen positive colonies (blue; arrow) were screened on QDO/X/A agar plates to interplay with pGBKT7-T1-ICD bait in old cDNA library of CMECs. **III:** Three candidate proteins that potentially interacted with TrkB-T1 were revealed via a blast homology analysis of the screened DNA sequence. The Prey A which is homology with Willin, also named FRMD6, was selected for further direct interaction analysis, as it was the only cytosol protein among the three screened candidates (regarding the other two candidates, Prey B was a membrane protein and Prey C was a nuclei protein). **IV:** No autoactivation activity of three screened Prey A, Prey B and Prey C with pGBKT7-T-BD plasmid in Y187 strain and Y2HGold cells yeast two-hybrid system. **V:** The interaction assay between 11 C-terminal amino acids of TrkB-T1 (Y2HGold containing pGBKT7-T1-C11 bait plasmid) and Willin (Prey A) revealed that TrkB-T1 recruits Willin as downstream effector via its intracellular domain of 11 amino acids of C-terminus. **VI:** 3D structure of Willin protein. P53+T: pGBKT-P53 vector + pGADT7-T vecrtor (positive control). Lam+T: pGBKT7-Lam + pGADT7-T vecrtor (negative control). BD: pGBKT7-BD vector (vector control). T1ICD: pGBKT7-T1-ICD vector (including TrkB-T1 intracellular domain). T1C11: pGBKT7-T1-C11 vector (including 11 C-terminal amino acids of TrkB-T1 intracellular domain).

**

**

**Figure S2** **BiFC assay reveals that the intracellular domain of the TrkB-T1 receptor interacted with Willin in live cells. I:** Schematic of BiFC for TrkB-T1 and Willin. **II:** The BiFC assay revealed a significantly higher fluorescent density was found in the TrkB-T1-VC155 and Willin-VN173 co-transfected 293T cell group but not in the TrkB-T1-VC155-, Willin-VN173-, VC-155-, and VN173-transfected 293T cell groups. The fluorescent density of TrkB-FL-VC155 and Willin-VN173 co-transfected 293T cell group was significantly lower than TrkB-T1-VC155 and Willin-VN173 co-transfected 293T cell group (*p*<0.05). (a) Representative image of BiFC assay in the individual group. (b) Semiquantitative analysis of (a) (*: *p*<0.05 vs. other groups). Scale bar=200 μm**. III:** The competitive BiFC strategy using TrkB-T1-FLAG-tag and Willin-HA-tag as competitors for TrkB-T1-VC155 and Willin-VN173, respectively, revealed that the fluorescence density decreased significantly upon transfection with the TrkB-T1-FLAG-tag expression vector or the Willin-HA-tag expression vector, respectively, with TrkB-T1-VC155+Willin-VN173 compared with the TrkB-T1-VC155+Willin-VN173 co-transfected group (*p*<0.05). In addition, the fluorescent density among TrkB-FL-VC155 + Willin-VN173 co-transfected-, TrkB-T1-VC155 + TrkB-T1-FLAG + Willin-VN173 co-transfected- and TrkB-T1-VC155 + Willin-HA + Willin-VN173 co-transfected- 293T cell groups was similar. (a) Representative image of the competitive BiFC assay for individual groups. (b) Semiquantitative analysis of (a) (*: *p*<0.05 vs. other groups). Bar = 200 μm.

**
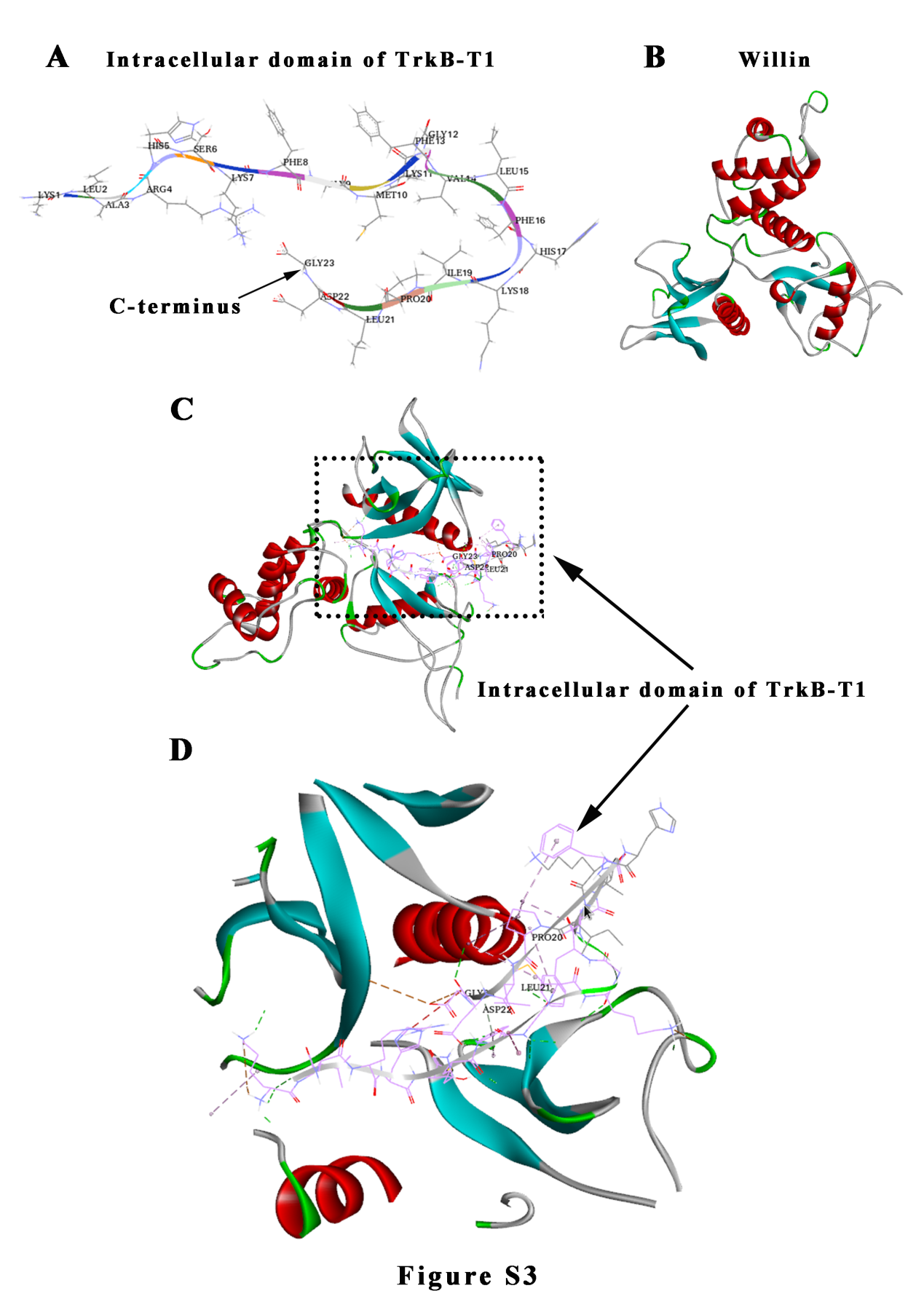
**

**Figure S3 Homology modeling analysis predicts that potential interaction between intracellular domain of TrkB-T1 and FERM domain of Willin. A**: The constructed 3D model of intracellular domain of TrkB-T1. **B**: The constructed 3D model of FERM domain of Willin (9-322 amino acids) using Ezrin as homologous model (top listed identity; sequence identity=22.8% and sequence similarity=48.6%). **C** and **D**: The results of ZDOCK and RDOCK analysis for interaction between intracellular domain of TrkB-T1 and FERM domain of Willin. It was revealed that the intracellular domain loop of TrkB-T1 is included in structural pocket of FERM domain of Willin and suggests that the intracellular domain of TrkB-T1 is able to interact with FERM domain of Willin. D: Enlargement of black dot line rectangle.

**Figure S4 Knocked down expression of Willin** **abrogates the promotion effect of the BDNF-TrkB-T1-Willin pathway in the TrkB-T1-transfected 293T cells.** **I:** (a) Transfection with si-Willin-1672, si-Willin-2095 and si-Willin-2255 knocked down the expression of Willin. si-Willin-2095 was applied in the subsequent experiments as it induced the strongest knockdown effect (*:*p*<0.05 vs. si-Willin-1672, Negative control and Lipo2000). (b) Representative images of different dosages of BDNF (80 ng/mL, 160 ng/mL and 240 ng/mL)-treated TrkB-T1-EGFP-transfected 293T cells when co-transfected with si-Willin-2095 under light and fluorescent microscopy. (c) Semiquantitative analysis of **I**(d)-(f) and **II**(b)-(d) (*p*>0.05). (d) A representative measurements of pseudopod migration under si-Willin-2095 transfection upon treatment with 80 ng/mL BDNF. (e) A representative measurement of pseudopod migration under si-Willin-2095 transfection upon treatment with 160 ng BDNF. (f) A representative measurement of pseudopod migration under si-Willin-2095 transfection upon treatment with 240 ng/mL BDNF. **II:** (a) Representative images of different dosages of BSA (80 ng/mL, 160 ng/mL and 240 ng/mL)-treated TrkB-T1-EGFP- transfected 293T cells when co-transfected with si-Willin-2095 under light and fluorescent microscopy. (b) A representative measurement of pseudopod migration under si-Willin-2095 transfection upon treatment with 80 ng/mL BSA. (c) A representative measurement of pseudopod migration under si-Willin-2095 transfection upon treatment with 160 ng/mL BSA. (d) A representative measurement of pseudopod migration under si-Willin-2095 transfection upon treatment with 240 ng/mL BSA. The time-lapse analysis demonstrated that knocked down expression of Willin using si-Willin-2095 interfering siRNA was able to abrogate the promotion effect of the BDNF-TrkB-T1-Willin pathway in the TrkB-T1-transfected 293T cells. Red rectangle indicates the measured cells. Bar = 40 μm. The results represent three independent experiments.


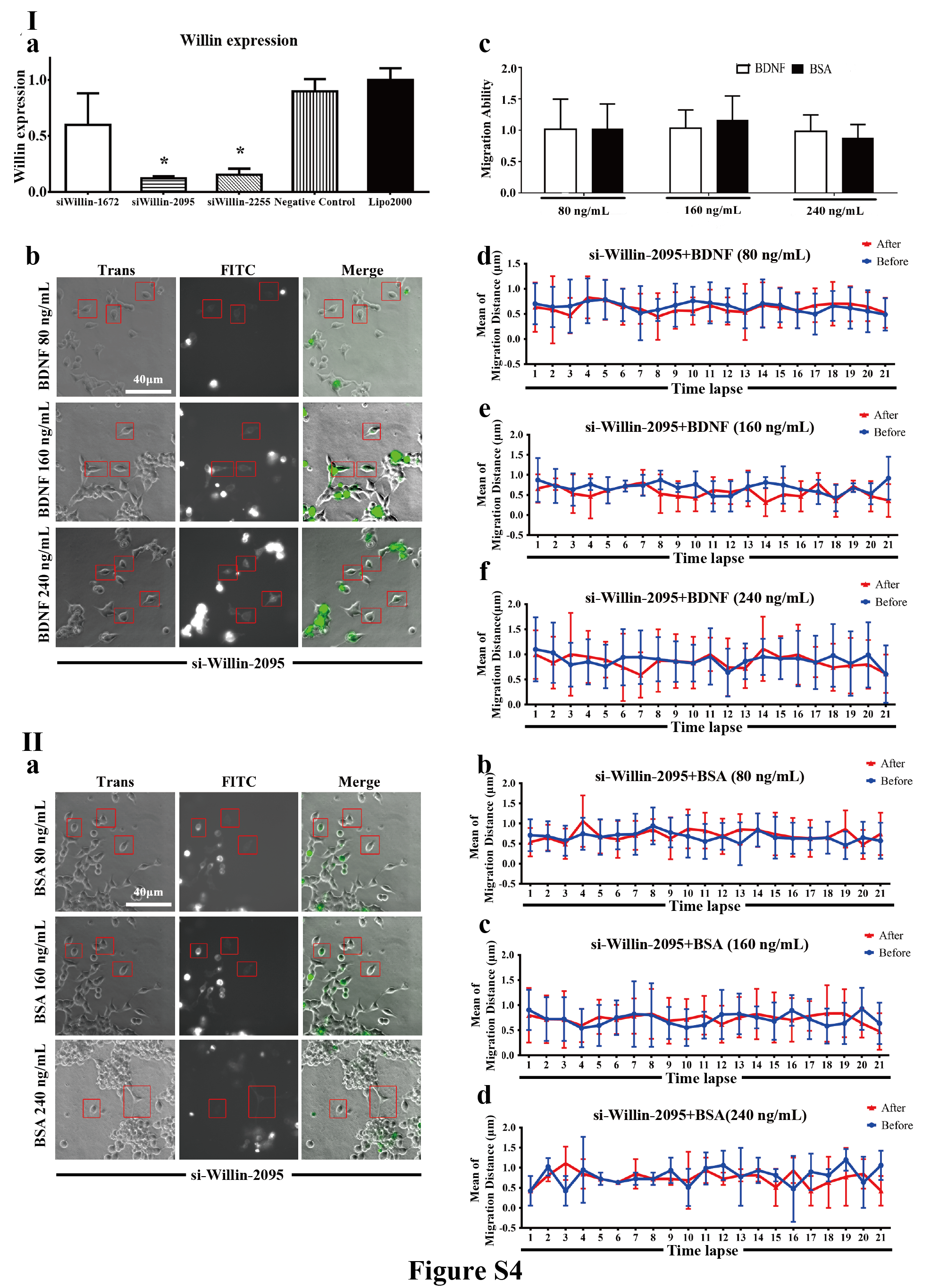

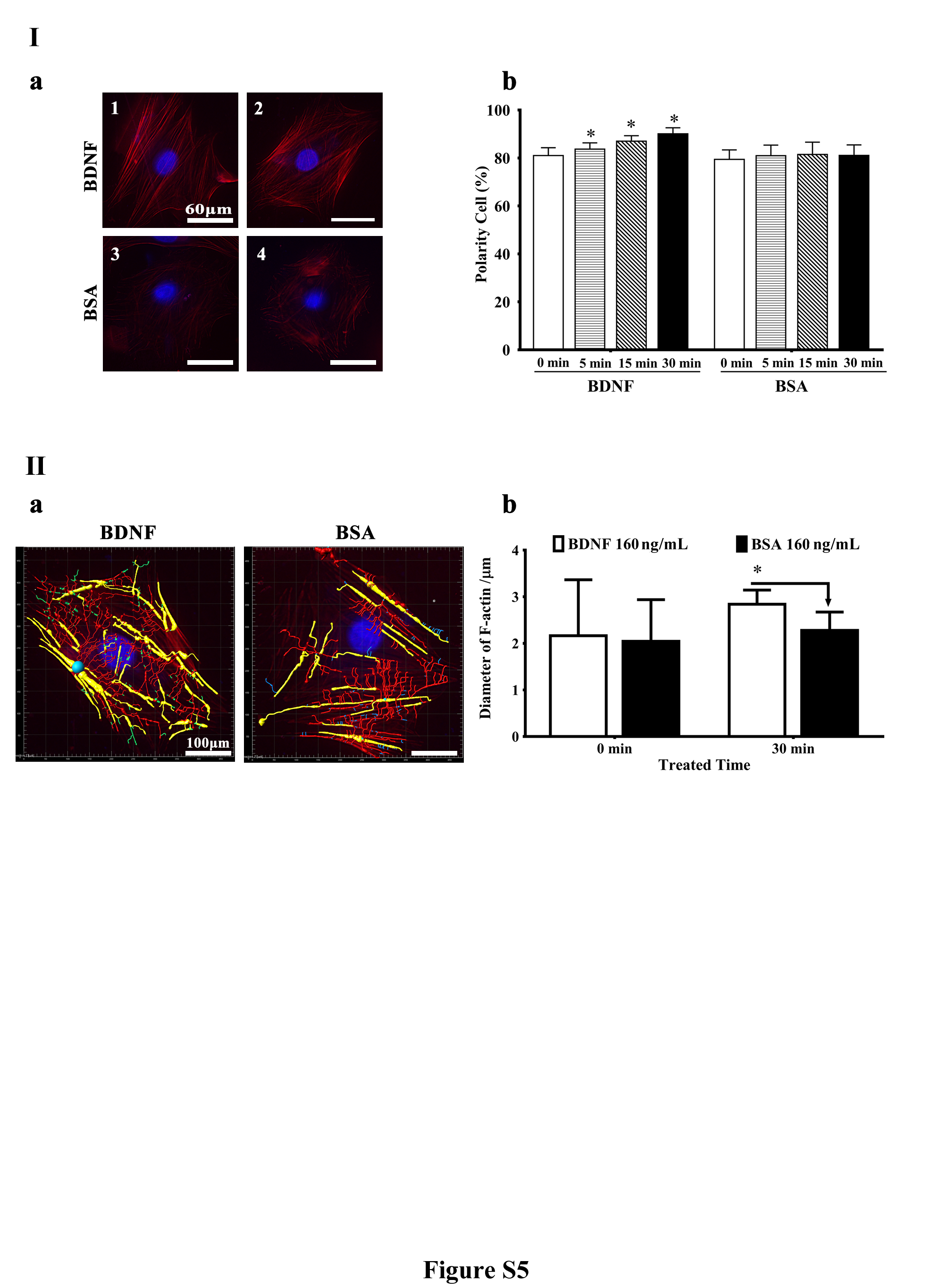


**Figure S5 BDNF-TrkB-T1-Willin pathway increases the polarity of actin and** **the polymerization of stress fiber. I:** (a) Representative images of the polarity of F-actin-positive fibers in non-BDNF-treated and BDNF (160 ng/mL)-treated old CMECs. (b) Semiquantitative analysis of polarized cells after BDNF (160 ng/mL) treatment compared with the BSA-treated control (*: *p*<0.05 vs. 0 min). Bar = 60 μm. **II:** (a) Representative images of the polymerization of stress fibers in BDNF (160 ng)-treated and non-BDNF-treated old CMECs. (b) Semiquantitative analysis of the diameter of the polymerization of stress fibers after BDNF (160 ng/mL) treatment. BDNF (160 ng/mL) treatment progressively increased the number of polarized cells at 5 min, 15 min and 30 min after BDNF treatment (*p*<0.05). In addition, the mean diameter of stress fibers in the BDNF-treated group was significantly larger than that of the BSA-treated group 30 min after BDNF treatment (*: *p*<0.05 vs. BSA). The results represent three independent experiments with three individual old CMECs.


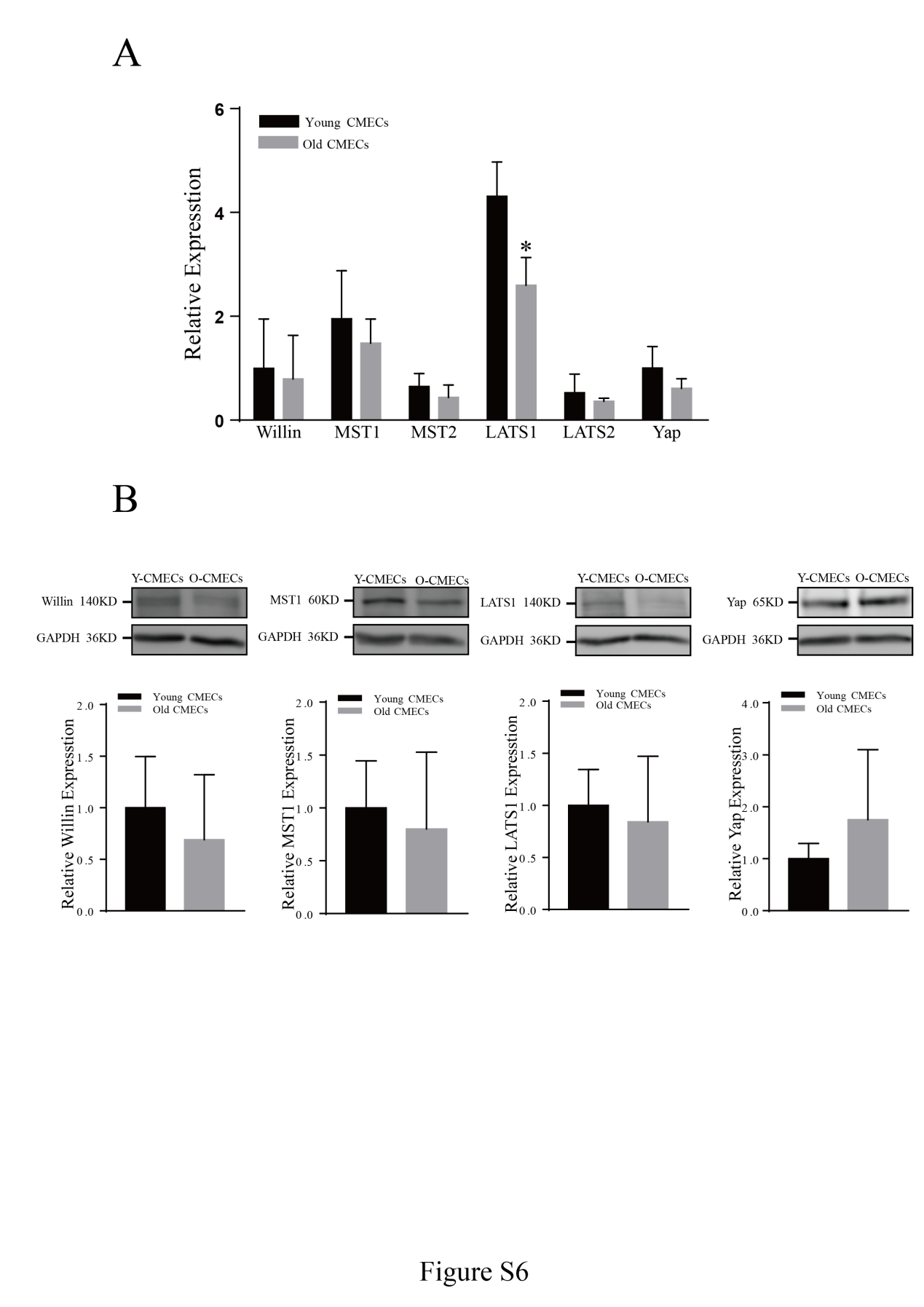


**Figure S6 The expressions of Willin and Key effectors of Hippo pathway in protein level are similar between young and old CMECs. A:** qPCR analysis revealed that the differences of the expression levels of *Willin, MST1, MST2, LATS2 and Yap* between young CMECs and old CMECs were not statistical significance (p>0.05), While, the expression of *LATS1* of young CMECs were significantly higher than that of old CMECs (p<0.05). **B:** The western blot results showed that the differences of expressions of Willin, MST1, LATS1 and Yap between young CMECs and Old CMECs were not statistical significance in protein level (p>0.05). The results represent three independent experiments.


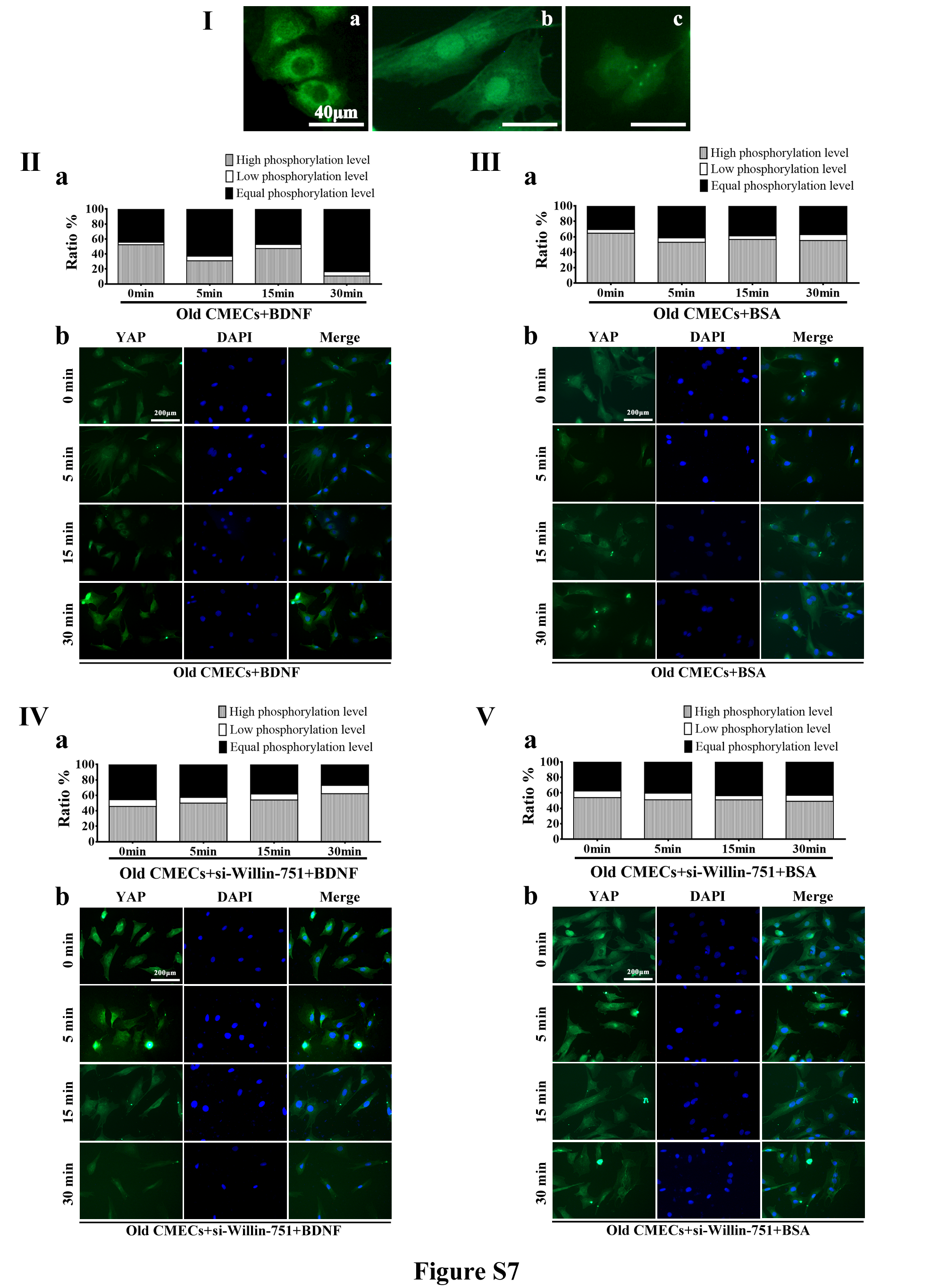


**Figure S7 Immunofluorescence staining further confirms that BDNF treatment induced translocation of Yap into the nuclei via the BDNF-TrkB-T1-Willin pathway. I:** A representative cell showing a density of Yap in the cytoplasm that is higher than in the nucleus (termed a high phosphorylation density cell) (a), is lower than in the nucleus (termed as a low phosphorylation density cell) (b) and is equal to the nucleus (termed an equal phosphorylation density cell) (c). Bar = 40 μm. **II:** (a) Semiquantitative analysis of the percentages of high-, low- and equal-phosphorylation level cells at 5-, 15- and 30-min after BDNF treatment. (b) Representative images of the different groups shown in (a). **III:** (a) Semiquantitative analysis of the percentages of high-, low- and equal-phosphorylation level cells at 5-, 15- and 30-min after BSA treatment. (b) Representative images of the different groups shown in (a). **IV:** (a) Semiquantitative analysis of the percentages of high-, low- and equal-phosphorylation level cells at 5-, 15- and 30-min after BDNF treatment in old CMECs that were pre-transfected with si-Willin-751 before BDNF treatment. (b) Representative images of the different groups shown in (a). **V:** (a) Semiquantitative analysis of the percentages of high-, low- and equal-phosphorylation level cells at 5-, 15- and 30-min after BSA treatment in old CMECs that were pre-transfected with si-Willin-751 before BSA treatment. (b) Representative images of the different groups shown in (a). BDNF treatment for 30 min incurred an increase in the percentage of equal-fluorescent intensity cells to approximately 83.65% compared to approximately 44.32% for the non-BDNF-treated cells. For the BSA control group treated for 30 min, the percentage of equal-fluorescent intensity cells was approximately 37.18%, which was similar to the non-BDNF-treated cells (approximately 30.59%). In addition, for the si-Willin-751 treated group, the percentage of equal-fluorescent intensity cells after BDNF treatment for 30 min (approximately 27.05%) was lower than that of the non-BDNF-treated cells (approximately 45.61%), while the percentage of equal-fluorescent intensity cells after BSA treatment for 30 min (approximately 43.14%) was similar to that of the non-BSA-treated cells (approximately 37.50%). Bar = 200 μm. The results represent three independent experiments with three individual old CMECs.

**Reference:**

Cai, D., Xaymardan, M., Holm, J. M., Zheng, J., Kizer, J. R., & Edelberg, J. M. (2003). Age-associated impairment in TNF-α cardioprotection from myocardial infarction. *American Journal of Physiology Heart & Circulatory Physiology, 285*(2), H463. https://doi.org/10.1152/ajpheart.00144.2003

Cao, L., Zhang, L., Chen, S., Yuan, Z., Liu, S., Shen, X., . . . Chan, Y. H. (2012). BDNF-mediated migration of cardiac microvascular endothelial cells is impaired during ageing. *Journal of Cellular & Molecular Medicine, 16*(12), 3105–3115. https://doi.org/10.1111/j.1582-4934.2012.01621.x

Hu, C. D., Chinenov, Y., & Kerppola, T. K. (2002). Visualization of interactions among bZIP and Rel family proteins in living cells using bimolecular fluorescence complementation. *Molecular Cell, 9*(4), 789-798. https://www.ncbi.nlm.nih.gov/pubmed/ 11983170

Kerppola, T. K. (2006). Design and implementation of bimolecular fluorescence complementation (BiFC) assays for the visualization of protein interactions in living cells. *Nature Protocols, 1*(3), 1278. https://doi.org/10.1038/nprot.2006.201

Shyu, Y. J., Liu, H., Deng, X., & Hu, C. D. (2006). Identification of new fluorescent protein fragments for bimolecular fluorescence complementation analysis under physiological conditions. *Biotechniques, 40*(1), 61-66. https://doi.org/10.1002/hed.20435

**Table S1: Predictive interacting amino acids between intracellular domain of TrkB-T1 and FERM domain of Willin via interface nonbond interaction**

| **Willin residue** | **TrkB-T1 intracellular domain residue** |
| --- | --- |
| ASP220:OD2 | LYS1:HT2 |
| ARG227:NE | GLY23:OCT2 |
| ARG301:NE | GLY23:OTC2 |
| ASP220:OD2 | LYS1:NZ |
| GLU83:OE2 | LYS11:NZ |
| GLN63:HE22 | LYS11:O |
| TYR69:HH | ASP22:OD2 |
| MET70:HN | LYS7:O |
| CYS298:SG | ASP22:O |
| THR300:HG1 | GLY9:O |
| GLN136:O | LYS1:HT1 |
| ASP220:OD1 | LYS1:HT3 |
| TYR225:OH | LYS1:HZ1 |
| ASP220:OD1 | LEU2:HN |
| GLN63:OE1 | GLY9:HN |
| MET70:O | GLY9:HN |
| GLU83:OE1  ARG301:NE  THR300:HG1  ARG301:HH22  TYR69  VAL138  CYS298 | LYS11:HZ3  ARG4:NE  PHE13  PHE8  PHE8:C,O;GLY9:N  LYS1  MET10 |
| CYS298 | PRO20 |
| PRO299  PRO299  LEU72  ARG303  PRO299 | VAL14  PRO20  PHE8  PHE13  PHE16 |

**Table S2: Predictive interacting amino acids between intracellular domain of TrkB-T1 and FERM domain of Willin via hydrogen bond**

| **Willin residue** | **Intracellular domain** | **Interaction Constituents** | **Distance of TrkB-T1 residue** |
| --- | --- | --- | --- |
| GLN63 | LYS11 | GLN63:HE22 - LYS11:O | 2.51 |
| TYR69 | ASP22 | TYR69:HH - ASP22:OD2 | 2.0884 |
| MET70 | LYS7 | MET70:HN - LYS7:O | 1.9416 |
| CYS298 | ASP22 | CYS298:SG - ASP22:O | 2.8286 |
| THR300 | GLY9 | THR300:HG1 - GLY9:O | 2.15 |
| GLN136 | LYS1 | LYS1:HT1 - GLN136:O | 1.8604 |
| ASP220 | LYS1 | LYS1:HT3 - ASP220:OD1 | 2.1597 |
| TYR225 | LYS1 | LYS1:HZ1 - TYR225:OH | 2.1642 |
| ASP220 | LEU2 | LEU2:HN - ASP220:OD1 | 2.2447 |
| GLN63 | GLY9 | GLY9:HN - GLN63:OE1 | 2.1322 |
| MET70 | GLY9 | GLY9:HN - MET70:O | 2.4691 |
| GLU83 | LYS11 | LYS11:HZ3 - GLU83:OE1 | 1.9735 |
